# The Suramin Derivative NF449 Interacts with the 5-fold Vertex of the Enterovirus A71 Capsid to Prevent Virus Attachment to PSGL-1 and Heparan Sulfate

**DOI:** 10.1371/journal.ppat.1005184

**Published:** 2015-10-02

**Authors:** Yorihiro Nishimura, Noel P. McLaughlin, Jieyan Pan, Sara Goldstein, Susan Hafenstein, Hiroyuki Shimizu, Jeffrey D. Winkler, Jeffrey M. Bergelson

**Affiliations:** 1 Division of Infectious Diseases, The Children’s Hospital of Philadelphia, Philadelphia, Pennsylvania, United States of America; 2 Department of Virology II, National Institute of Infectious Diseases, Musashimurayama-shi, Tokyo, Japan; 3 Department of Chemistry, University of Pennsylvania, Philadelphia, Pennsylvania, United States of America; 4 Department of Microbiology and Immunology, Pennsylvania State University College of Medicine, Hershey, Pennsylvania, United States of America; 5 Department of Pediatrics, University of Pennsylvania Perelman School of Medicine, Philadelphia, Pennsylvania, United States of America; The Scripps Research Institute, UNITED STATES

## Abstract

NF449, a sulfated compound derived from the antiparasitic drug suramin, was previously reported to inhibit infection by enterovirus A71 (EV-A71). In the current work, we found that NF449 inhibits virus attachment to target cells, and specifically blocks virus interaction with two identified receptors—the P-selectin ligand, PSGL-1, and heparan sulfate glycosaminoglycan—with no effect on virus binding to a third receptor, the scavenger receptor SCARB2. We also examined a number of commercially available suramin analogues, and newly synthesized derivatives of NF449; among these, NF110 and NM16, like NF449, inhibited virus attachment at submicromolar concentrations. PSGL-1 and heparan sulfate, but not SCARB2, are both sulfated molecules, and their interaction with EV-A71 is thought to involve positively charged capsid residues, including a conserved lysine at VP1-244, near the icosahedral 5-fold vertex. We found that mutation of VP1-244 resulted in resistance to NF449, suggesting that this residue is involved in NF449 interaction with the virus capsid. Consistent with this idea, NF449 and NF110 prevented virus interaction with monoclonal antibody MA28-7, which specifically recognizes an epitope overlapping VP1-244 at the 5-fold vertex. Based on these observations we propose that NF449 and related compounds compete with sulfated receptor molecules for a binding site at the 5-fold vertex of the EV-A71 capsid.

## Author Summary

Enterovirus A71 is epidemic in the Asia-Pacific region, and has been responsible for thousands of cases of fatal neurological disease in young children. There are no specific therapies available. We previously identified NF449 as a compound with anti-EV-A71 activity, although its mechanism of action was uncertain. In the current work we found that NF449 and related molecules prevent virus attachment both to PSGL-1, a receptor molecule important for virus interaction with white blood cells, and to heparan sulfate, a receptor that may be important for virus interaction with a variety of other cell types. In contrast, we found that NF449 had no effect on virus attachment to another proposed receptor, SCARB2. We also found that NF449 and related compounds interact with a specific site on the viral capsid, remote from the binding site for another major receptor, SCARB2. Our work provides information that may facilitate development of improved antiviral compounds that block the attachment of EV-A71 to cellular receptors.

## Introduction

Enterovirus A71 (EV-A71, formerly named enterovirus 71) is a non-enveloped single-stranded RNA virus that belongs to the enterovirus A group of human picornaviruses (for a general review of EV-A71 see [1]). EV-A71 most often causes a mild childhood illness, hand-foot-mouth disease. However, some infected children suffer severe complications, which include flaccid paralysis, brainstem encephalitis, and cardiorespiratory failure. Although EV-A71 was first isolated in California, its major impact is now felt in the Asia-Pacific region. In an ongoing epidemic in mainland China, nearly 7 million cases of EV-A71 disease have occurred since 2008, with more than 80,000 severe cases and over 2,400 deaths [2]. Several inactivated vaccine candidates show promising efficacy and safety profiles [3–5]; however, it is not clear when EV-A71 vaccines will be introduced for widespread use or whether they will provide protection against multiple EV-A71 genotypes [6]. At present, there are no specific therapies for EV-A71: treatment is entirely supportive, with severe cases requiring intensive management in critical care units [7–9].

One potential target for antiviral therapies is the interaction between EV-A71 and receptor molecules on host cells. EV-A71 has been reported to bind to several different receptors, including scavenger receptor class B member 2 (SCARB2) [10], P-selectin glycoprotein ligand-1 (PSGL-1, a molecule primarily expressed on blood cells) [11], and heparan sulfate glycosaminoglycans [12]; virus interactions with annexin II [13], vimentin [14], and nucleolin [15] have also been reported to promote infection, although their importance is less clear. We have shown that EV-A71 interaction with PSGL-1 on leukocytes requires the presence of sulfated tyrosine residues near the N-terminus of PSGL-1 [16], and depends on two highly conserved lysine residues, VP1-244K and VP1-242K, near the 5-fold vertex of the viral capsid [17]. Another residue near the 5-fold vertex, VP1-145, determines whether or not a particular isolate binds PSGL-1 (with G or Q in isolates that bind PSGL-1, E in those that do not), by influencing the orientation of VP1-244K [17]. In addition to their role in PSGL-1 binding, the positively-charged lysine residues at the 5-fold vertex have been proposed—although not yet confirmed—to be important for virus interaction with heparan sulfate [12].

We previously identified NF449, (4, 4', 4'', 4‴- [carbonylbis[imino- 5, 1, 3- benzenetriylbis(carbonylimino)]]tetrakis- 1, 3- benzenedisulfonic acid), as an inhibitor of EV-A71 infection in a screen of a compound library [18]; NF449 inhibited EV-A71 infection, but not poliovirus infection, and showed no detectable cellular toxicity. Inhibition was seen when NF449 was added at the start of infection, but not after 2 hrs, suggesting that the drug acts at an early stage in the virus life cycle. We isolated an NF449 escape mutant that had undergone two mutations within the viral capsid—one involving VP1-244K, the other involving VP1-98E, a neighboring residue at the 5-fold vertex. Based on these observations, we hypothesized that NF449, which contains sulfonated aromatic rings closely resembling sulfotyrosines, binds to EV-A71 near the 5-fold vertex and interferes with virus attachment to PSGL-1 and other sulfated cellular receptors.

## Results

### NF449 specifically inhibits EV-A71 attachment to RD cells

**Fig 1:**
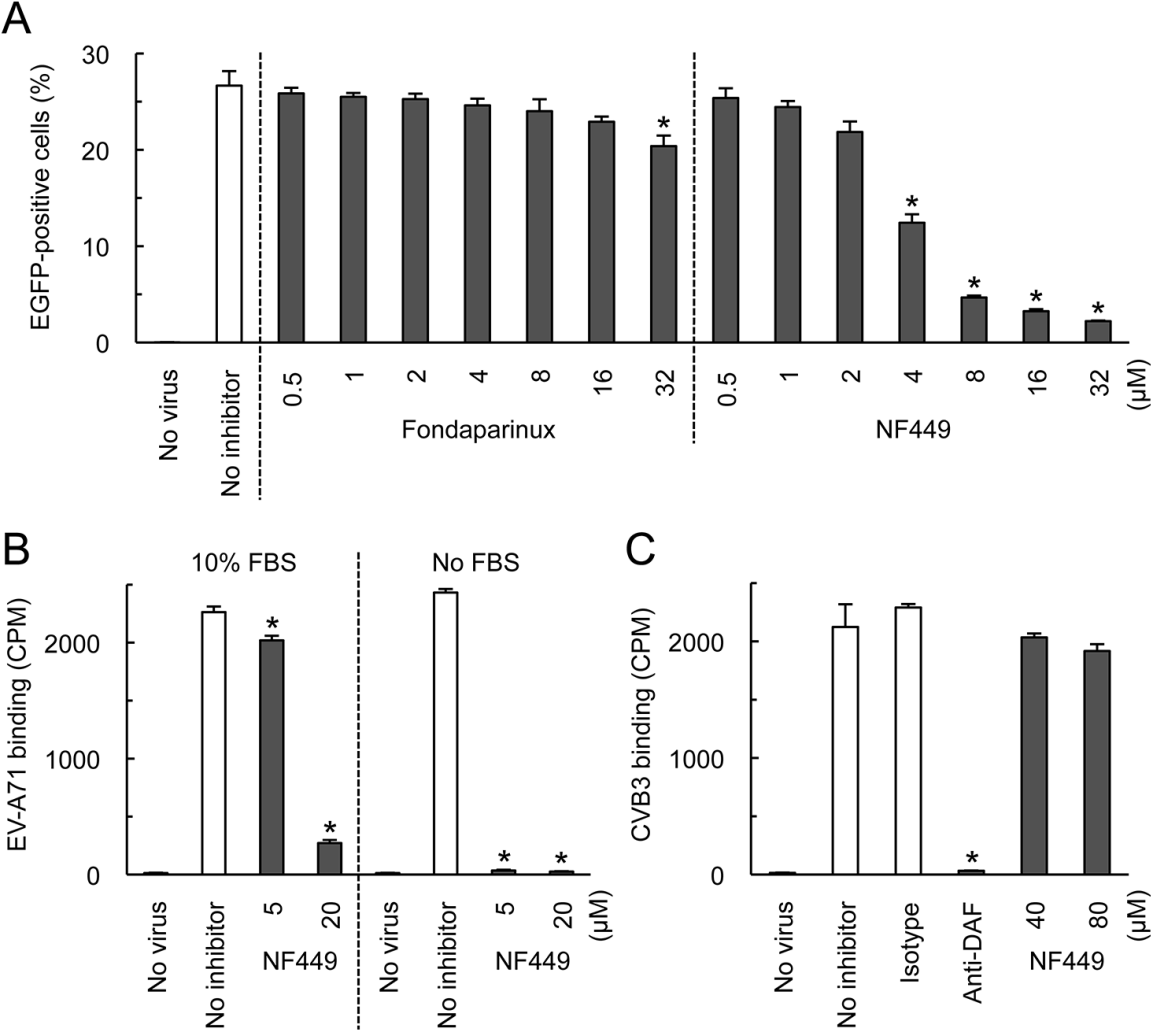
NF449 inhibits EV-A71 interactions with RD cells. (A) RD cell monolayers were exposed to EGFP-expressing EV-A71-1095 in the presence of NF449 or the control compound fondaparinux at the indicated concentrations, then incubated at 37°C for 16 hr. Infected cells were identified by flow cytometry to detect EGFP expression. (B) ^35^S-labeled EV-A71-1095 was exposed to NF449 in medium with or without 10% FBS, then attachment to RD monolayers was measured as described in Materials and Methods. (C) HeLa cell monolayers were exposed to ^35^S-labeled CVB3-RD in the presence of NF449, the anti-DAF antibody IF7, or an isotype-matched control antibody, and attachment was measured. Results are indicated as the mean and S.D. for triplicate samples. Asterisks indicate *P* < 0.01 compared to results with the no inhibitor control.

**Fig 2:**
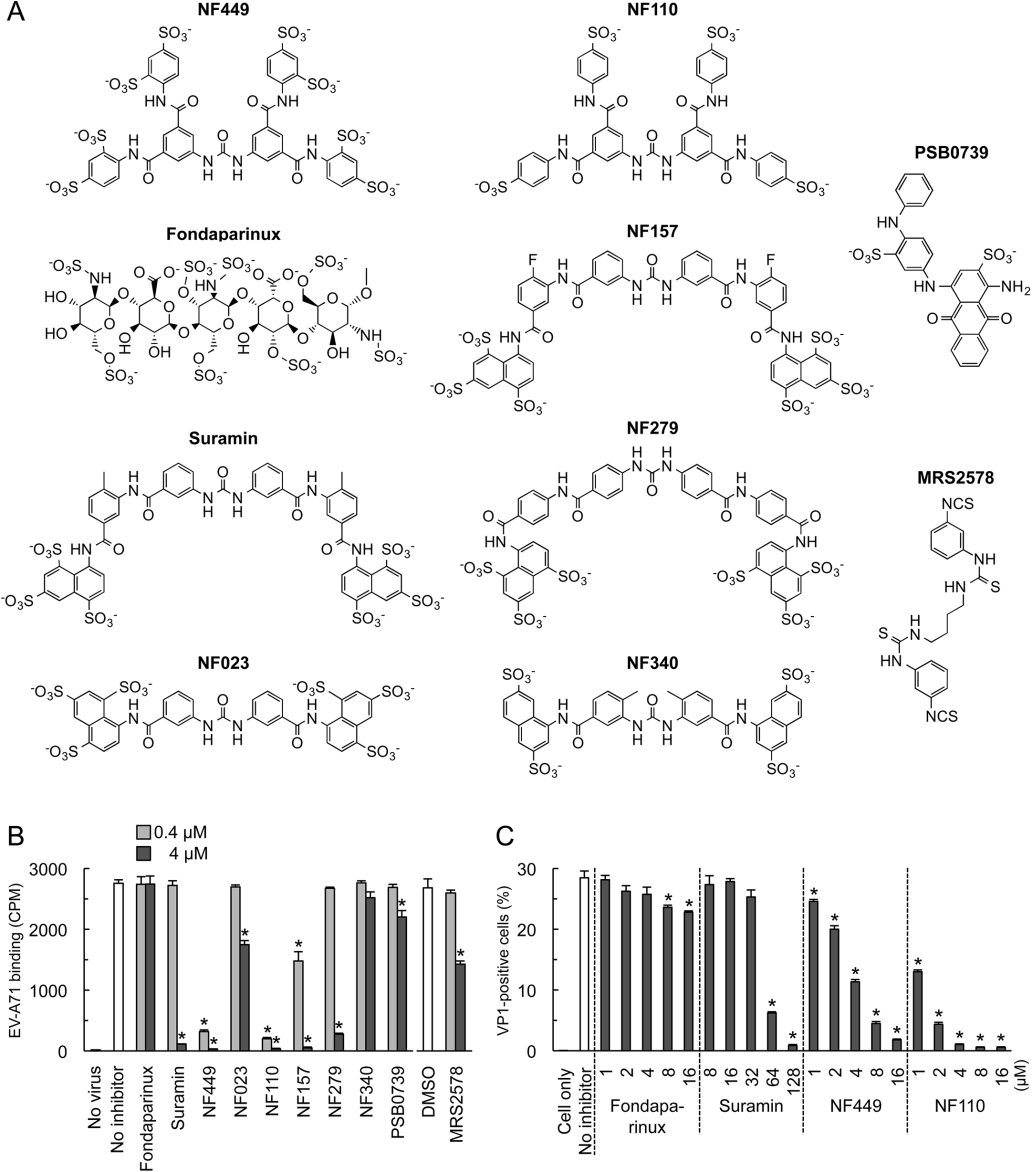
Inhibition of EV-A71 attachment and infection by commercially-available NF449 analogues. (A) Structures of the tested compounds. (B) ^35^S-labeled EV-A71-1095 was exposed to compounds at 0.4 or 4 μM, then virus attachment to RD monolayers was measured. (C) RD monolayers were exposed to EV-A71-1095 in the presence of inhibitors at the indicated concentrations. After 16 hrs at 37°C, infected cells were stained to detect intracellular VP1, and analyzed by flow cytometry. Results are indicated as the mean and S.D. of triplicate samples. Asterisks indicate *P* < 0.01 compared to the no inhibitor control.

We first confirmed the earlier observation [18] that NF449 inhibits EV-A71 infection. EV-A71-1095-EGFP, a PSGL-1-binding isolate engineered to express enhanced green fluorescent protein (EGFP) in infected cells, was incubated with NF449 or with control medium, then added to RD cells. After 16 hours, infection was measured by flow cytometry to detect GFP expression. NF449 had a strong inhibitory effect at 4–32 μM (Fig 1A); in contrast, a control compound, the synthetic heparin analogue fondaparinux, whose molecular weight, net negative charge, and degree of sulfation are similar to those of NF449 (structures are shown in Fig 2A), had little effect.

NF449 markedly inhibited attachment of ^35^S-labeled EV-A71-1095 to RD cells (Fig 1B, left panel), at a concentration similar to that required for inhibition of infection; in contrast, NF449 had no effect on attachment of radiolabeled coxsackievirus B3, a control enterovirus that binds to receptors distinct from those recognized by EV-A71 [19](Fig 1C). These results suggest that NF449 interferes specifically with EV-A71 attachment to the cell surface—by interacting with the virus, with receptors on the cell surface, or with both. NF449 is derived from suramin [20], a compound known to bind extensively to serum proteins [21]. We observed that NF449 was much more effective in inhibiting virus attachment in the absence of serum (Fig 1B, right panel, and see below). To assess the avidity of compounds for the virus and/or receptors, without complications introduced by the presence of serum proteins, further binding experiments (but not experiments testing inhibition of infection) were performed in serum-free medium.

### NF449 and NF110 are more effective inhibitors than a variety of other suramin analogues

To begin to understand the structural basis of NF449's antiviral activity, we compared the inhibitory effects of NF449 to those of a number of commercially-available compounds with related structures (Fig 2A). At 4 μM concentration several of the commercial compounds—including NF449 and suramin—effectively blocked virus attachment (Fig 2B); however, when tested at 0.4 μM, only NF449 and NF110 showed a marked inhibitory effect. None of the compounds showed cytotoxicity at the doses used to inhibit virus attachment (S1 Fig). When we measured the effect of NF110 and NF449 on virus replication (experiments performed in serum-containing medium), NF110 inhibited replication of EV-A71-1095 in RD cells at a somewhat lower concentration than did NF449 [NF110 50% inhibitory concentration (IC_50_), 1 μM; NF449 IC_50_, 4 μM] (Fig 2C); both NF449 and NF110 inhibited infection more effectively than the parent compound, suramin (IC_50_ 32–64 μM). NF110 is very similar in structure to NF449, but it has only 4 sulfonate groups, exclusively at the para positions of the aromatic rings, whereas NF449 has 8 sulfonate groups, at both ortho and para positions. The results thus suggested that activity was not strictly related to the number of sulfonate groups or the net negative charge, but more likely related to to the specific orientation of sulfonate groups and to other features of the molecule.

### Small changes in the NF110 structure have marked effects on antiviral activity

**Fig 3:**
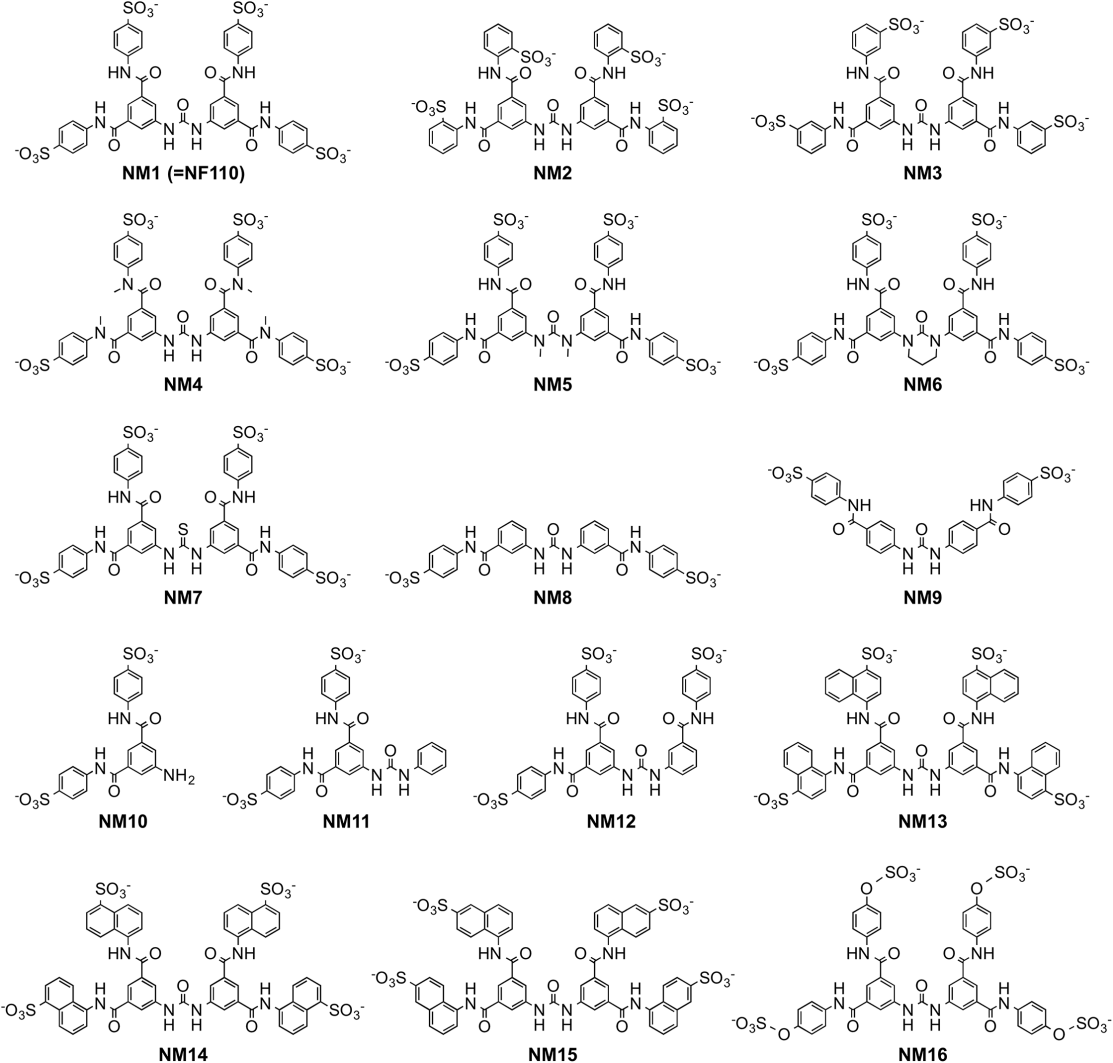
Structures of newly-synthesized NF449 analogues. Compounds NM2-16 are new. NM1 was resynthesized, but is identical to NF110.

With the idea of identifying a more effective compound than NF110, we synthesized a number of new analogues (Fig 3). Our working hypothesis was that NF110 binds to the capsid surface, with electrostatic interactions between its sulfonate residues and capsid lysines, stabilized by hydrophobic and hydrogen bonding interactions involving other parts of the molecule. Based on this, we changed the orientation, number, and spacing of the sulfonate groups (NM1-3 and NM13-15); we altered potential hydrophobic and hydrogen bonding properties by alkylating amide and urea NH bonds (NM4 and 5) and by replacing the urea oxygen with sulfur (NM7); and we reduced the molecule’s flexibility by confining the urea moiety in a 6-membered ring (NM6). We also produced an NF110-like molecule in which the peripheral aromatic rings were modified to resemble more closely a sulfotyrosine moiety (NM16).

**Fig 4:**
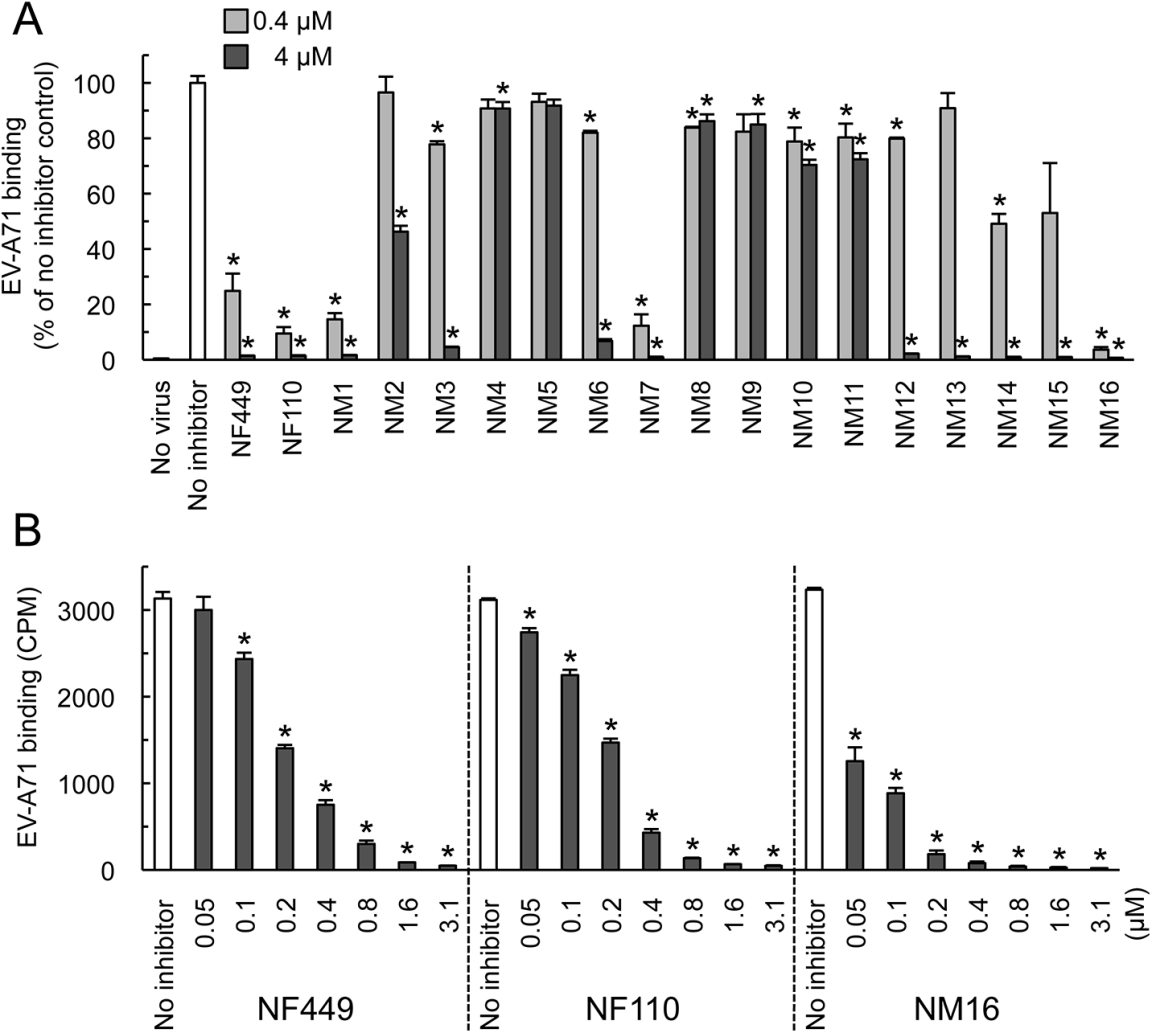
Inhibition of EV-A71 attachment by newly-synthesized NF449 analogues. (A) Attachment of ^35^S-labeled EV-A71-1095 to RD cells in the presence of compounds at 0.4 or 4 μM. Results from two experiments, one testing NM1-11 and one testing NM12-16, are combined, and are normalized to results for the no inhibitor control in each experiment. (B) Virus attachment to RD monolayers in the presence of NF449, NF110, and NM16. Results are indicated as the mean and S.D. for triplicate samples. Asterisks indicate *P* < 0.01 compared to the no inhibitor control.

When these new compounds were tested for their effect on virus attachment, several showed no activity even at 4 μM, and at 0.4 μM only NM7 and NM16 showed activity comparable to that of NF110 and NF449 (Fig 4A). At lower concentrations, NM16 was more active than NF110 and NF449 (Fig 4B), reducing attachment of EV-A71-1095 to RD cells by 50% even at 0.05 μM. None of the new compounds showed cytotoxicity at the doses used to inhibit virus attachment and infection (S1 Fig).

### NF449 and NF110 inhibit PSGL-1-dependent infection of Jurkat cells

**Fig 5:**
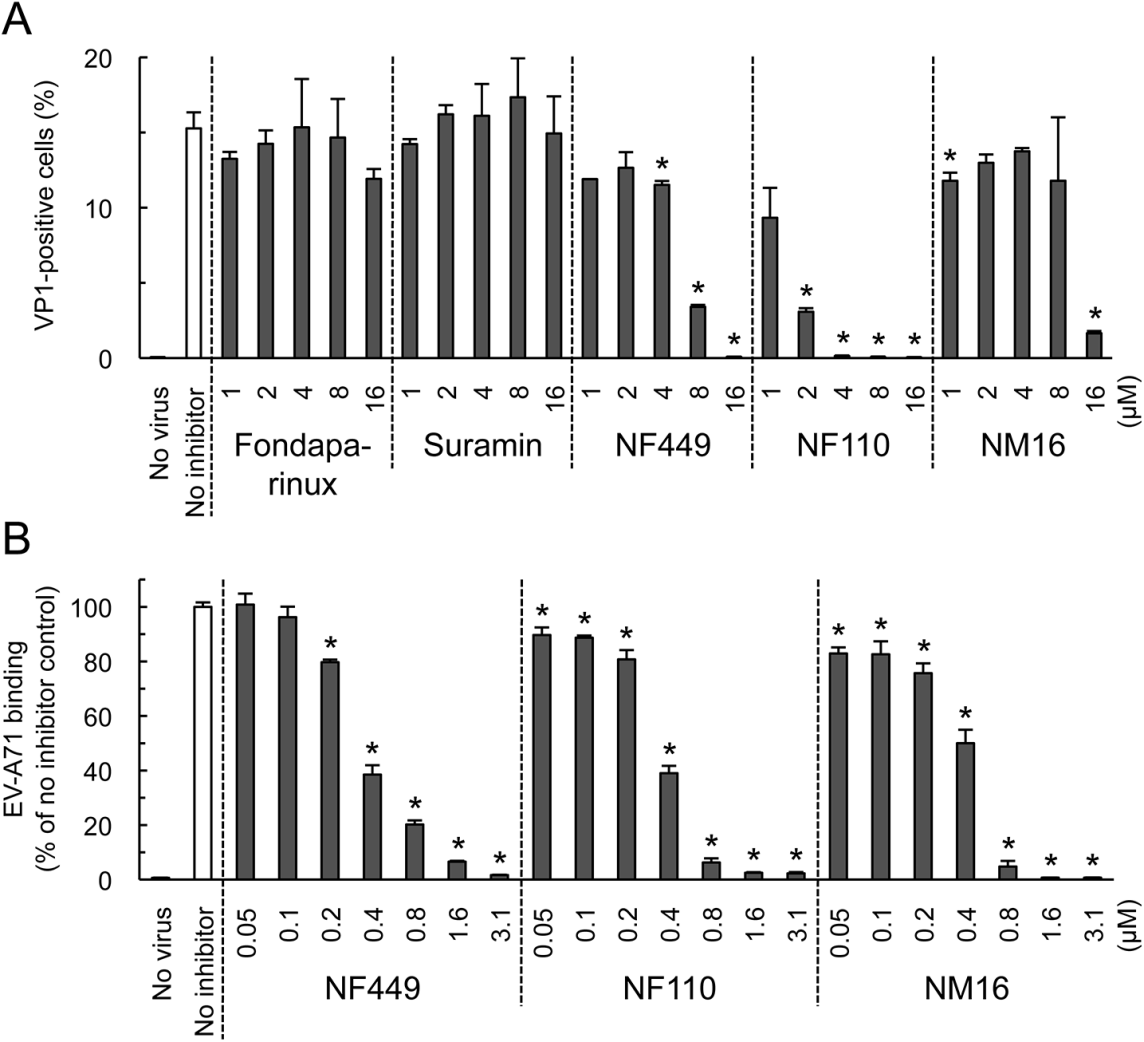
NF449, NF110, and NM16 inhibit virus interactions with Jurkat cells. (A) Infection by EV-A71-1095, in the presence of inhibitors, measured by staining for VP1 expression. (B) Inhibition of virus attachment by NF449, NF110, and NM16. Results are indicated as the mean and S.D. for triplicate samples. Asterisks indicate *P* < 0.01 compared to the no inhibitor control.

Some, but not all, EV-A71 isolates bind to PSGL-1 [11, 17], a receptor molecule expressed largely on haematopoietic cells, and not expressed on RD cells. These PSGL-1-binding (PB) isolates, which include EV-A71-1095, infect Jurkat T-cells in a PSGL-1-dependent manner [11, 17]. We found that NF110 and NF449 inhibited infection of Jurkat cells by EV-A71-1095 (Fig 5A), at concentrations slightly higher than those required to block infection of RD cells (Fig 2C) whereas suramin and fondaparinux had no effect (Fig 5A). NM16 also blocked infection of Jurkat cells, but was less effective than NF110 and NF449. However, in virus binding assays, performed in the absence of serum, NF449, NF110, and NM16 all inhibited attachment of radiolabeled EV-A71-1095 to Jurkat cells at sub-micromolar concentrations (Fig 5B).

### NF449 and NF110 inhibit infection by PSGL-1-nonbinding (non-PB) isolates, as well as by PB isolates

**Fig 6:**
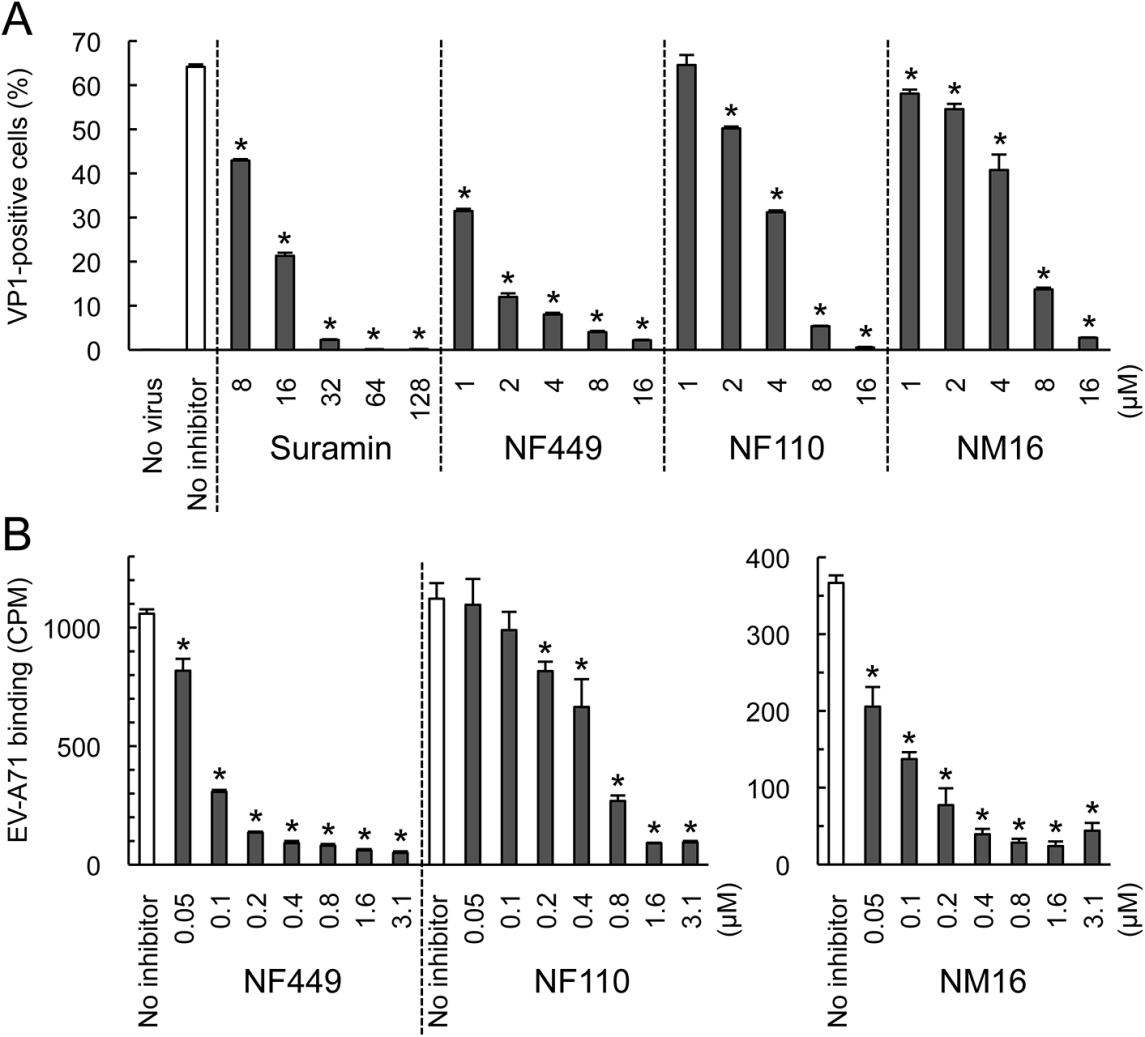
NF449, NF110, and NM16 inhibit interaction with RD cells of a PSGL-1-nonbinding EV-A71 isolate. (A) Infection by EV-A71-02363 in the presence of inhibitors, measured by staining for VP1 expression (B) Attachment of ^35^S-labeled EV-A71-02363 in the presence of NF449 and NF110 (left), and NM16 (right). Results are indicated as the mean and S.D. for triplicate samples. Asterisks indicate *P* < 0.01 compared to the no inhibitor control.

NF449 was previously shown to inhibit infection by a number of EV-A71 isolates [18], although one isolate (BrCr) showed partial resistance. We subsequently determined that, unlike the other isolates tested, BrCr does not bind PSGL-1 [11, 17]. This raised the possibility that non-PB viruses are relatively resistant to NF449 and related compounds. To test this, we examined another non-PB isolate, EV-A71-02363. Infection of RD cells by EV-A71-02363 was inhibited by NF449, NF110, and NM16 (Fig 6A), and all three compounds inhibited attachment of radiolabeled EV-A71-02363 (Fig 6B).

However, whereas NF110 was more effective than NF449 in inhibiting infection by EV-A71-1095, NF449 was more effective than NF110 against EV-A71-02363. These results suggest that at least some non-PB isolates are sensitive to inhibition by NF449, NF110, and NM16, although particular inhibitors may more effective against particular viral isolates.

### NF449 and NF110 block EV-A71 binding to PSGL-1 and heparin, but not binding to SCARB2

**Fig 7:**
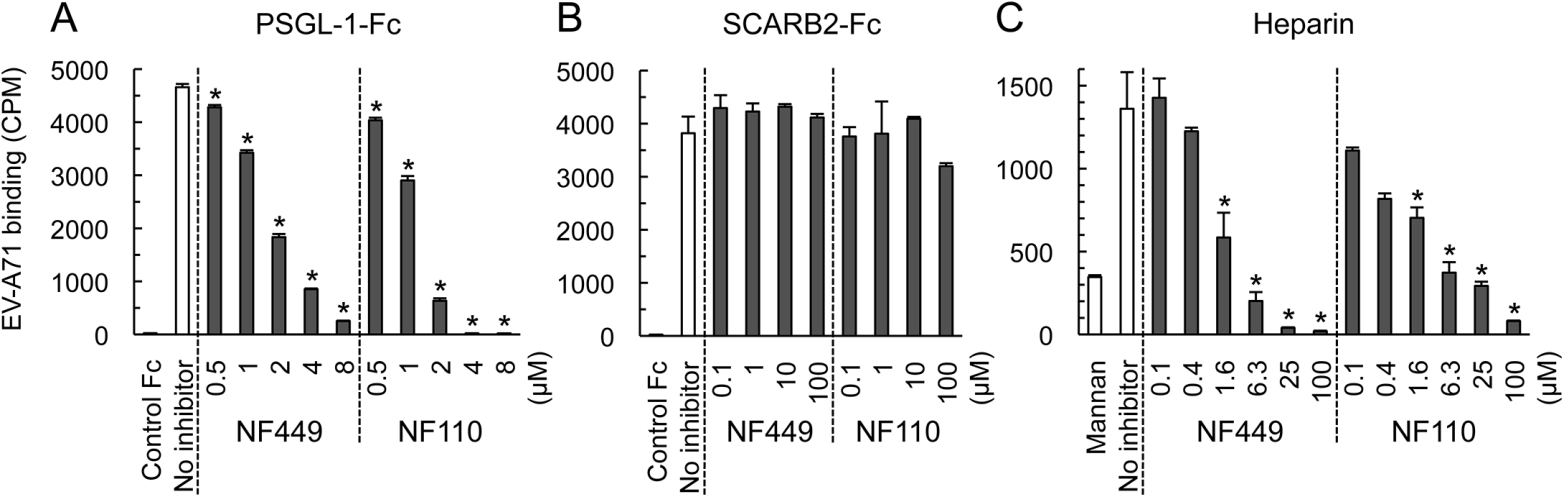
NF449 and NF110 inhibit EV-A71 attachment to PSGL-1 and heparin, but not SCARB2. (A) PSGL-1-Fc fusion protein or a control fusion protein fixed to beads were incubated with ^35^S-labeled EV-A71-1095 in the presence of inhibitors, and attachment was measured as described in Materials and Methods. (B) Attachment to SCARB2-Fc (in this experiment 8 x 10^3^ CPM was used). (C) Attachment to heparin- or to control mannan-coated beads. Results are indicated as the mean and S.D. for triplicate samples. Asterisks indicate *P* < 0.01 compared to the no inhibitor control.

EV-A71-1095 bound to SCARB2-Fc and PSGL-1-Fc fusion proteins fixed to magnetic beads (Fig 7A and 7B), but not to a control Fc fusion protein. EV-A71-1095 also bound beads coated with heparin (a form of heparan sulfate, Fig 7C), but not to beads coated with mannan, a nonsulfonated polysaccharide. NF449 and NF110 blocked virus binding to both PSGL-1 and heparin at low micromolar concentrations, but had no effect on virus binding to SCARB2.

### NF449 and NF110 block the interaction of a monoclonal antibody with the 5-fold vertex

**Fig 8:**
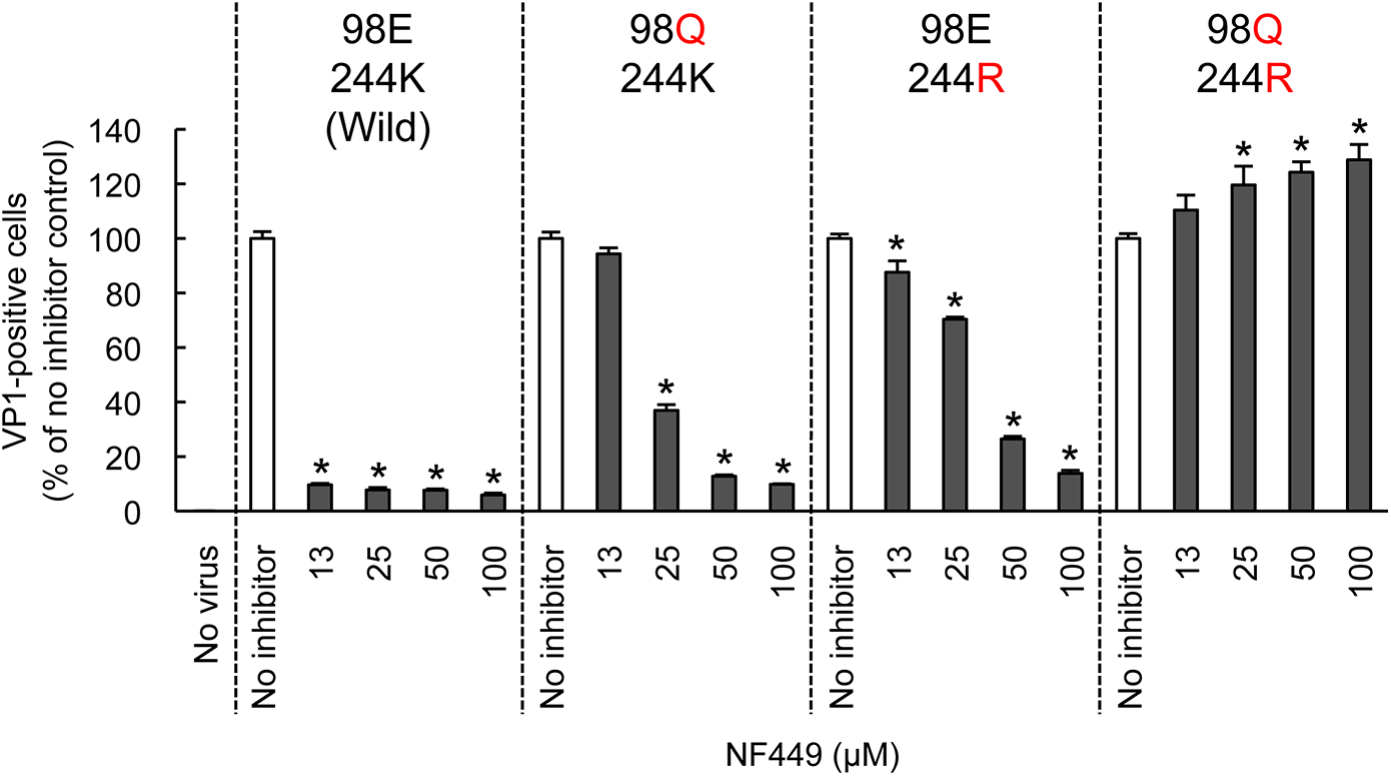
Mutations at the 5-fold capsid vertex reduce the protective effect of NF449. Wild-type EV-A71-1095 and VP1 mutants were exposed to NF449 for 1 h, applied to RD cell monolayers and incubated at 37°C for 16 h. Cells were then stained to detect intracellular VP1 and analyzed by flow cytometry. Results are indicated as the mean and S.D. of triplicate samples. Asterisks indicate *P* < 0.01 compared to no inhibitor control.

Both PSGL-1 and heparan sulfate are sulfonated molecules, which may interact directly with positively-charged lysine residues clustered near the 5-fold capsid vertex. In contrast, current evidence suggests that SCARB2, which is not sulfonated, binds in or near the viral canyon, at some distance from the 5-fold axis [22]. To confirm that residues near the 5-fold axis influence EV-A71 susceptibility to NF449, we introduced specific mutations into EV-A71-1095, and determined whether the mutant viruses were inhibited by NF449 (Fig 8). NF449 protected RD cells from infection by wild-type virus, but showed a reduced effect against viruses with an arginine (R) residue replacing lysine (K) at VP1-244, or with glutamine (Q) replacing glutamate (E) at VP1-98; the virus with both mutations was not inhibited by NF449 at any concentration tested. The results indicate that VP1-244, which is critical for virus attachment to PSGL-1, and the nearby residue VP1-98, are both important for inhibition by NF449, and they suggest that NF449 interferes with receptor access to a site near the 5-fold vertex.

**Fig 9:**
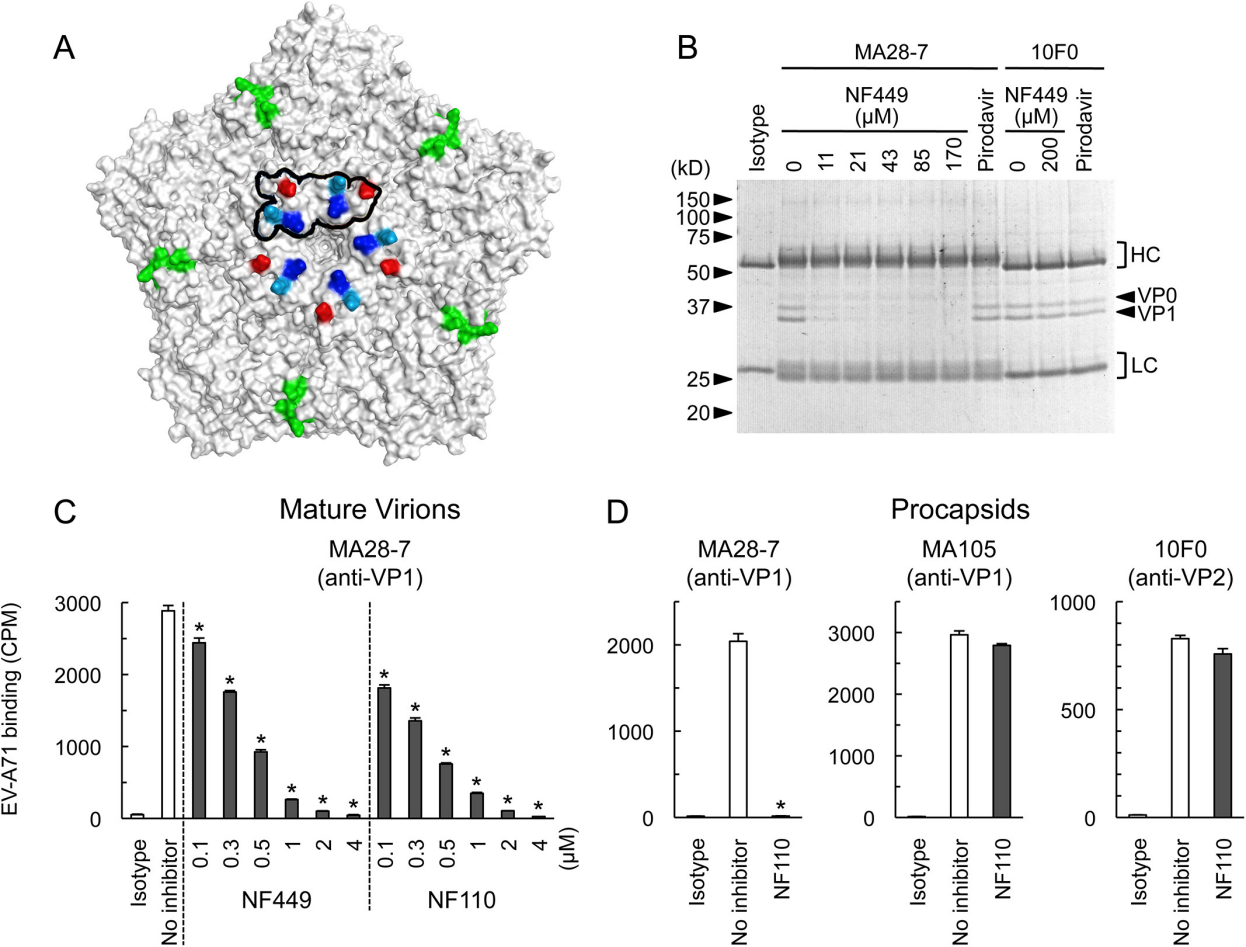
NF449 and NF110 specifically prevent attachment of a monoclonal antibody to the viral 5-fold vertex. (A) An EV-A71 pentamer shown with the 5-fold vertex at the center. VP1-98 (red) was mutated in an NF449 escape mutant. VP1-242K (light blue) is involved in virus interaction with PSGL-1. VP1-244K (dark blue) is implicated both in virus interactions with both PSGL-1 and NF449. The MA28-7 footprint, as determined by cryo-electronmicroscopy, is outlined in black. The VP2 epitope recognized by mAb 10F0 is indicated in green. (B) NF449 inhibits immunoprecipitation of EV-A71-1095 by MA28-7 but not by 10F0. EV-A71-1095 concentrated from supernatants of infected cells was incubated with MA28-7 or 10F0 fixed to Protein G beads, in the presence of NF449 at the indicated concentrations, or 200 μM Pirodavir; beads were washed, and immunoprecipitated proteins were examined on Coomassie-stained gels. Molecular weight markers are shown at the left. Arrows at the right indicate capsid proteins VP0 and VP1, and brackets indicate antibody heavy (HC) and light chains (LC). (C) NF449 and NF110 inhibit attachment of MA28-7 to mature virions. Purified ^35^S-labeled mature virions were incubated with MA28-7-coated beads, in the presence of inhibitors as indicated. (D) NF110 inhibits attachment of MA28-7, but not 10F0 or MA105, to procapsids. Purified ^35^S-labeled procapsids were incubated with monoclonal antibodies fixed to Protein G beads, in the presence of 20 μM NF110. Results are indicated as the mean and S.D. for triplicate samples. Asterisks indicate *P* < 0.01 compared to the no inhibitor control.

A neutralizing monoclonal antibody raised against EV-A71-1095, MA28-7, binds specifically to the 5-fold vertex, with a footprint (defined by cryo-electron microscopy [23]) encompassing VP1-98E and VP1-244K (Fig 9A). MA28-7 immunoprecipitated EV-A71-1095 capsids from solution (Fig 9B) as did another EV-A71 antibody, 10F0, which recognizes an epitope within VP2, remote from the 5-fold axis [24]. NF449 inhibited precipitation of virus by MA28-7, but not by 10F0. Pirodavir, an antiviral compound that is likely to interact with the viral canyon [25, 26] had no effect on virus interaction with either antibody. These results suggest that NF449 specifically competes with attachment of MA28-7 to its epitope at the 5-fold vertex.

When we repeated these experiments with radiolabeled virus, which is purified by sedimentation in sucrose gradients, interaction of 160S mature particles with MA28-7 was inhibited by sub-micromolar concentrations of NF449 and NF110 (Fig 9C). Surprisingly, we found that both 10F0 and MA105, an antibody that recognizes an undefined epitope within VP1, failed to immunoprecipitate purified 160S mature particles (S2 Fig) although (Fig 9D, S2 Fig), they did effectively precipitate purified 80S procapsids. NF110 inhibited interaction of MA28-7 with procapsids (Fig 9D), just as it inhibited MA28-7 interaction with mature virions. In contrast, NF110 had no effect on procapsid interaction with 10F0 or MA105. Taken together, the results support the idea that NF449 and NF110 interact with the 5-fold vertex and block virus attachment to MA28-7.

## Discussion

The results we report here indicate that NF449 and related compounds interact with the 5-fold capsid vertex to block attachment of enterovirus A71 to receptors on the cell surface. NF449 inhibited attachment of radiolabeled EV-A71 both to RD cells and to Jurkat cells, and it inhibited attachment both to PSGL-1 and to heparin, a form of heparan sulfate. Both PSGL-1 [17] and heparan sulfate [12] have been proposed to interact with positively-charged lysine residues at the 5-fold vertex of the EV-A71 capsid, and our results suggest that NF449 inhibits receptor interaction by binding to an overlapping site. NF449’s inhibitory effect was markedly reduced by mutation of residues near the 5-fold vertex, VP1-98E as well as VP1-244K— a highly conserved residue to be important for virus interaction with PSGL-1. Further, we found that NF449 blocked attachment of a monoclonal antibody specific for the 5-fold vertex.

While this work was in progress, other investigators reported that suramin, NF449’s parent compound, inhibits EV-A71 infection of RD cells and blocks virus attachment to the RD cell surface [12, 27, 28]; suramin was further shown to interact with the EV-A71 capsid (as determined by STD NMR), and to inhibit EV-A71 replication in mice and in non-human primates [28]. Because it has previously been approved for use in humans, suramin will likely be tested for clinical efficacy against EV-A71 infection. However, suramin has a number of toxic effects [29], some of them dose-related [30], and treatment of infected children may require development of new agents with greater antiviral activity at lower doses. We found that NF449, NF110, and NM16 consistently inhibited EV-A71 infection at concentrations lower than those required for inhibition by suramin.

We attempted to modify the NF110 structure to produce a more potent antiviral compound: although NM16, the tetra-sulfotyrosine analog was somewhat more effective than NF110 and NF449 in preventing EV-A71-1095 attachment to RD cells, none of the other novel analogs showed greater potency than the parent compounds. Nonetheless, our work provides several insights into structure-activity relationships among this series of compounds. First, relocation of the sulfonate groups to other positions in the NF110 structure (NM2, NM3) led to partial loss of activity, and truncated structures—lacking one or more of the aromatic rings and accompanying sulfonate groups (NM8-NM12)—had markedly reduced activity. These results suggest that antiviral activity depends on sulfonate groups in specific orientations. Further, activity was lost when N-methyl groups replaced the N-H groups of the amide (NM4) or urea (NM5) functionalities, which suggests that the hydrogen bonding capability of secondary amide groups is also likely to be important.

VP1-244K and the adjacent lysine residue at VP1-242 are present in virtually all EV-A71 isolates [17], suggesting that a concentration of positive charge at the 5-fold vertex serves an important function in the virus life cycle, and that it may provide an attractive target for antiviral agents. We do not yet know precisely how NF110, NF449, or other sulfonated inhibitors bind to the virus surface. However, using a molecular docking program, we found that NF449’s size, and the spacing between its sulfonate groups, may permit it to bridge multiple VP1-244K and -242K residues around the 5-fold vertex (S3 Fig). The capacity to bind multiple lysine residues simultaneously may be important for activity, and may explain why the more active compounds have sulfate or sulfonate residues at particular positions. Particular inhibitors may vary in efficacy in blocking particular virus-cell interactions, so that understanding structure-function relationships will require consideration of how each compound interacts with the surface of a particular virus isolate, as well as how effectively it blocks interaction with a particular receptor.

Taken together, our results provide evidence that the 5-fold vertex is important for PSGL-1-dependent virus attachment to leukocytes as well as for attachment to cells that do not express PSGL-1. Further, they identify a series of prototype compounds that bind the 5-fold vertex to block EV-A71 interaction with specific cell surface receptors, most likely because their sulfonate residues mimic tyrosine sulfate moieties near the N-terminus of PSGL-1, and sulfated/sulfonated components of heparan sulfate or other cell surface molecules. We believe that these results provide insights that—combined with ongoing structural studies of NF449 analogs bound to virus—will facilitate the design of potent antiviral compounds.

## Materials and Methods

### Cells

RD cells obtained from the US Centers for Disease Control were maintained in DMEM (Life Technologies) supplemented with 10% heat-inactivated FBS (HI-FBS). Jurkat cells were obtained from the Riken Cell Bank and cultured in DMEM without phenol red (Life Technologies) supplemented with 10% heat-inactivated fetal bovine serum. HeLa cells obtained from the ATCC were maintained in MEM (Sigma-Aldrich) supplemented with 5% HI-FBS, 1x non-essential amino acids (Life Technologies) and 100 U/ml penicillin and 100 μg/ml streptomycin (Life Technologies).

### Viruses

EV-A71-1095 (PB) and EV-A71-02363 (non-PB) were used. EV-A71-02363 was generated from an infectious viral cDNA clone (pBREV71-02363-KE) as described previously [17]. Both viruses were propagated in RD cells. Viral titers were determined by a microtitration assay using 96-well plates and RD cells as previously described [31]. Briefly, 10 wells were used for each viral dilution and the viral titers were expressed as 50% cell culture infectious dose (CCID_50_). Coxsackievirus B3 (RD strain) [32] was used as a control.

### Construction of EV-A71-EGFP

EV-A71 modified to express EGFP in infected cells (pBREV71-1095-EGFP-EG) was generated as described previously [10] with modifications. EV-A71 cDNA (pBREV71-1095-EG, [17]) and pEGFP (Clontech) were used as templates for overlap extension PCR. DNA encoding EGFP was amplified, with the addition of an EV-A71 protease 2A recognition sequence (AITTLGS; 2A cleaves between TL and GS) at the 3'-end, and inserted into EV-A71 VP4, between residues 3 and 4 (MGS-QVS…). Protease 2A releases EGFP (with the N-terminal extension MGS and the C-terminal extension AITTL) as well as VP4 (GSQVS…); the initial methionine of VP4 is normally cleaved to permit myristylation of the N-terminal glycine residue [33].

### cDNA clones encoding mutant viruses

Mutations were introduced into the pBREV71-1095-EG plasmid [17] by site directed mutagenesis using PCR. For mutagenesis from E to Q at VP1-98, the primers 5'-ccctcttcaaggcacaaccaacccgaatgg-3' and 5'-gtgccttgaagagggaggtctatctctcca-3' were used. For mutagenesis from K to R at VP1-244, the primers 5'-tcgaaatcccgttacccattagtggtcaggattt-3' and 5'-tgggtaacgggatttcgaggtccctacagtccgca-3' were used. The plasmid with the VP1-E98Q mutation was named pBREV71-1095-QG. The plasmid with the VP1-K244R mutation was named pBREV71-1095-EG-K244R. The plasmid with both VP1-E98Q and VP1-K244R mutations was named pBREV71-1095-QG-K244R.

### Antibodies and recombinant proteins

The anti-EV-A71 mAbs MA28-7 (mouse IgG_1_) [11] and MA105 (mouse IgG_2b_) [23], were generated from mice immunized with EV-A71-1095. The anti-EV-A71 mAb 10F0 (IgG_1_) was purchased from Abcam. Anti-DAF antibody (IF7, mouse IgG_2b_) [34] was used to inhibit Coxsackievirus B3 binding to HeLa cells. For negative controls, mouse IgG_1_ (MOPC-21) and IgG_2b_ (MOPC-141) were purchased from Biolegend and Sigma-Aldrich, respectively. Soluble recombinant forms of human proteins fused to the Fc region of human IgG_1_ (PSGL-1-Fc, SCARB2-Fc, and CTLA-4-Fc) were purchased from R&D Systems. CTLA-4-Fc was used as a negative control Fc protein.

### Compounds

Suramin hexasodium salt, NF023, NF110, NF157, NF279, NF340, NF449, PSB0739, and MRS2578 were purchased from Tocris Bioscience. Fondaparinux sodium (Arixtra) was purchased from GlaxoSmithKline. Pirodavir was a kind gift from Dr. John Lambert, Biota Pharmaceuticals. MRS2578 and Pirodavir were dissolved in DMSO and used for the experiments.

### Chemical synthesis

The synthesis of all compounds was based on the work of Kassack, et. al. [35]. Compounds were purified by silica gel chromatography, and purity was assessed by HPLC or NMR (^1^H and ^13^C). Structures were analyzed by NMR, infrared, and high resolution mass spectrometry. All compounds were synthesized as sodium salts, but Na+ ions are not indicated in figures. Experimental details (S1 Appendix) for all new compounds are included in the Supplementary Material

### Evaluation of drug cytotoxicity

Drug cytotoxicity was evaluated by measuring ATP as a marker of metabolically active cells. RD cells (5x10^3^) were cultured with diluted drugs (total 25 μl/well, triplicate) in a 384-well plate (Corning) at 37°C for 16 h. ATP levels were measured using a CellTiter-Glo 2.0 luminescent cell viability assay kit (Promega) according to the manufacturer’s instructions.

### EV-A71-EGFP infection assays

EV-A71-EGFP (10^5^ CCID_50_/100 μl, 400 μl) was incubated with diluted inhibitors (400 μl) in DMEM supplemented with 10% HI-FBS (800 μl total) on ice for 1 h. The virus-inhibitor mixture (200 μl: 10^5^ CCID_50_) was then added to RD cells (5x10^4^ cells per well in a 48-well plate: 2 CCID_50_/cell) in triplicate. The plate was incubated at 4°C with gentle agitation for 1 h, then incubated in a CO_2_ incubator at 37°C for 16 h. The cells were trypsinized, fixed with 4% paraformaldehyde and analyzed using a FACSCalibur (Becton-Dickinson).

### EV-A71 infection assays

EV-A71-1095 or EV-A71-02363 (4x10^5^ CCID_50_/100 μl, 400 μl) was incubated with diluted inhibitors (400 μl) in DMEM supplemented with 10% HI-FBS (800 μl total) on ice for 1 h. The virus-inhibitor mixture (200 μl; 4x10^5^ CCID_50_) was added to RD cells (5x10^4^ cells per well in a 48-well plate; 8 CCID_50_/cell) in triplicate. The plate was incubated at 4°C for 1h with gentle agitation, then incubated in a CO_2_ incubator at 37°C for 16 h. RD cells were trypsinized, fixed, permeabilized with 1X permeabilization buffer (eBioscience), stained with MA105 conjugated with Alexafluor 488 [11] and analyzed by FACSCalibur. For Jurkat cell infection, EV-A71-1095 (4x10^5^ CCID_50_/100 μl, 400 μl) was incubated with diluted inhibitors (400 μl) in DMEM without phenol red supplemented with 10% HI-FBS (800 μl total) on ice for 1 h. The virus-inhibitor mixture (800 μl: 1.6x10^6^ CCID_50_) was added to Jurkat cells (2x10^5^ cells: 8 CCID_50_/cell) in a 1.5 ml tube and incubated on ice for 1 h. The Jurkat cells with the virus and inhibitor (200 μl: 5x10^4^ cells; 8 CCID_50_/cell) were added to a well in a 96-well plate in triplicate and incubated in a CO_2_ incubator at 37°C for 16 h, then washed, fixed, permeabilized, stained and analyzed without trypsinization.

### ^35^S labeling of EV-A71

Radiolabeled virus was prepared as described with minor modifications [36]. Briefly, HeLa cells (1x10^7^ cells) were infected with EV-A71-1095 (2x10^9^ CCID_50_). For non-PB virus, RD cells (5x10^6^ cells) were incubated with EV-A71-02363 (8x10^9^ CCID_50_ for 45 min at room temperature, and then washed and incubated at 37°C in methionine/cysteine-free medium (Life Technologies). After 5 h, the medium was replaced with 4 ml of methionine/cysteine-free medium containing 100 μCi of ^35^S-methionine/cysteine per ml and incubation continued overnight. Cells were lysed by freezing and thawing three times, and then lysates were made in 0.5% Triton X-100 and clarified by centrifugation. Sodium dodecyl sulfate (1%) was added, and then the virus was pelleted through a 30% sucrose cushion using an SW55Ti rotor (Beckman Coulter) (45,000 rpm, 16°C, 90 min). The virus pellet was resuspended in phosphate buffered saline without calcium or magnesium (PBS) and purified by sedimentation through 15 to 35% sucrose gradients using a SW55Ti rotor (45,000 rpm, 16°C, 60 min). Twenty-four 0.2 ml fractions were collected from the top of each gradient and 5 microliter samples were analyzed for radioactivity: procapsids were detected in fractions 12–16 (peak, fraction 13) and mature virions in fractions 18–23 (peak, fraction 19).

### ^35^S-EV-A71–cell binding inhibition assay

^35^S-labeled EV-A71 (^35^S-EV-A71) (5x10^3^ CPM, unless indicated) was incubated with diluted inhibitors in DMEM without FBS on ice for 1 h. RD cells (10^5^ cells per well in a 48-well plate) were washed once with DMEM without FBS and incubated with the mixture of ^35^S-EV-A71 and inhibitors with gentle agitation at 4°C for 1.5 h. Jurkat cells were washed once with DMEM without FBS and incubated with the mixture of ^35^S-labeled EV-A71 and inhibitors on ice for 1 h. Unbound virus was removed with at least two washes with DMEM without FBS. RD cells were lysed with Solvable detergent (Perkin-Elmer), and cell-bound radioactivity was assessed. ^35^S-CVB3 was prepared as described [32].

### ^35^S-EV-A71 receptor binding inhibition assay

The EV-A71-receptor binding assay was performed as in [11], with minor modifications. Briefly, 5 μl of Dynabeads protein G (Life Technologies) and 0.5 μg of chimeric Fc proteins were diluted in 100 μl of DMEM without phenol red supplemented with 0.01% Tween 20 (DMEM-T) and incubated using a rotary mixer for 1 h at 4°C. The beads were washed once. ^35^S-EV-A71 (5x10^3^ CPM, unless indicated) were incubated with diluted inhibitors in DMEM-T on ice for 1 h. Then the virus-inhibitor mixture was added to Dynabeads protein G with chimeric Fc proteins and incubated using a rotary mixer for 1 h at 4°C. Unbound virus was removed with two washes with DMEM-T. Then Dynabeads-bound radioactivity was assessed. To detect EV-A71 binding to heparin, heparin-agarose (Sigma-Aldrich) was used. As a sulfonate-negative control, mannan-agarose (Sigma-Aldrich) was used. For ^35^S-EV-A71–antibody binding inhibition assays, anti-EV-A71 mAbs (0.5 μg) were bound to Dynabeads instead of chimeric Fc proteins.

### Preparation of unlabeled EV-A71 for receptor binding assay

RD cells infected with EV-A71-1095 were lysed by freezing and thawing three times. The medium with virus and cell debris was clarified by centrifugation. Virus in the supernatant was pelleted through a 30% sucrose cushion using an SW28 rotor (Beckman Coulter) (27,000 rpm, 16°C, 3h). The virus pellet (mixture of procapsid and mature virion) was resuspended in PBS and used for receptor binding inhibition assays.

### Unlabeled EV-A71 receptor binding inhibition assay

Dynabeads protein G and chimeric Fc proteins were prepared as described above. Viruses purified by ultracentrifugation (0.5 μg of VP1 protein in SDS-PAGE analysis) and inhibitors were incubated in 100 μl of DMEM-T for 1 h on ice. Then the virus-inhibitor mixture was added to Dynabeads protein G bound to chimeric Fc proteins and incubated using a rotary mixer for 1 h at 4°C. We washed the beads and subjected the immunoprecipitates to 12.5% SDS-PAGE. For western blotting, proteins were transferred onto nitrocellulose membranes and blotted with anti-EV-A71 VP1 mAb MA105. For EV-A71-antibody binding inhibition assay, anti-EV-A71 mAbs were used instead of chimeric Fc proteins to bind to Dynabeads.

### Molecular docking

The Molecular Operating Environment (MOE) Software Package [37] was used to draw the molecular surface of EV-A71 (PDB 4AED) [38] and to simulate molecular docking of NF449. The Site Finder function was used to identify potential binding sites around the 5-fold vertex, then the Pharmacophore setting was used to impose the constraint that at least one sulfonate group of NF449 must interact with at least one VP1-244K residue. Fifty-six potential docking sites were identified. For each of two possible conformations of NF449, using the GBVI/WSA dG scoring function for binding free energy, we selected three sites with the highest (negative) S scores to represent in S3 Fig.

### Statistics

All infection and binding assays were carried out in triplicate, and the mean values were compared using Student’s *t*-test (two-tailed). *P* values <0.01 were considered statistically significant.

### Accession numbers

The following sequences are deposited in GenBank: EV-A71-1095 (AB550332); EV-A71-02363 (AB747375); and pBREV71-1095-EGFP-EG (LC053680).

## Supporting Information

S1 Fig Cytotoxicity of inhibitor compounds. RD cells were cultured in the presence of diluted drugs in triplicate for 16 h. Cell viability was then determined by measuring a luminescent signal, which indicated the amount of ATP present, as described in Materials and Methods.
(PDF)

S2 Fig Recognition of capsids by monoclonal antibodies. (A) Virus immunoprecipitated by MA28-7 or 10F0 was resolved by SDS-PAGE, and either stained with Coomassie blue (left panel) or transferred to membranes and stained with mouse monoclonal antibody MA105 (specific for VP1) followed by horseradish peroxidase-conjugated anti-mouse IgG antibody (right panel). (B) Aliquots of pooled gradient fractions containing ^35^S-labeled Procapsids or Mature virions were immunoprecipitated with MA28-7, MA105, or 10F0, and precipitates were analyzed by scintillation counting. Similar results were obtained in two independent experiments.
(TIF)

S3 Fig Possible models for interaction of NF449 at the 5-fold vertex of EV-A71. Docking sites were simulated under the constraint that one of the sulfonate groups of NF449 must interact with at least one VP1-244K residue (yellow arrowheads). The six sites with highest scores were selected from a total of 56 possible sites identified by MOE software. On the virus surface, VP1-98 is colored red, VP1-244K dark blue, and VP1-242K light blue. NF449 carbon atoms are grey, with nitrogen blue, sulfur yellow, and oxygen red.
(TIF)

S1 Appendix Methods for synthesis of compounds NM1-16. (DOCX)
