# Supplementary figures and images for: The Suramin Derivative NF449 Interacts with the 5-fold Vertex of the Enterovirus A71 Capsid to Prevent Virus Attachment to PSGL-1 and Heparan Sulfate
Source: PLoS Pathog. 2015 Oct 2;11(10):e1005184. doi: 10.1371/journal.ppat.1005184 (PMC4592248; doi:10.1371/journal.ppat.1005184)

Luminescence (relative light units)

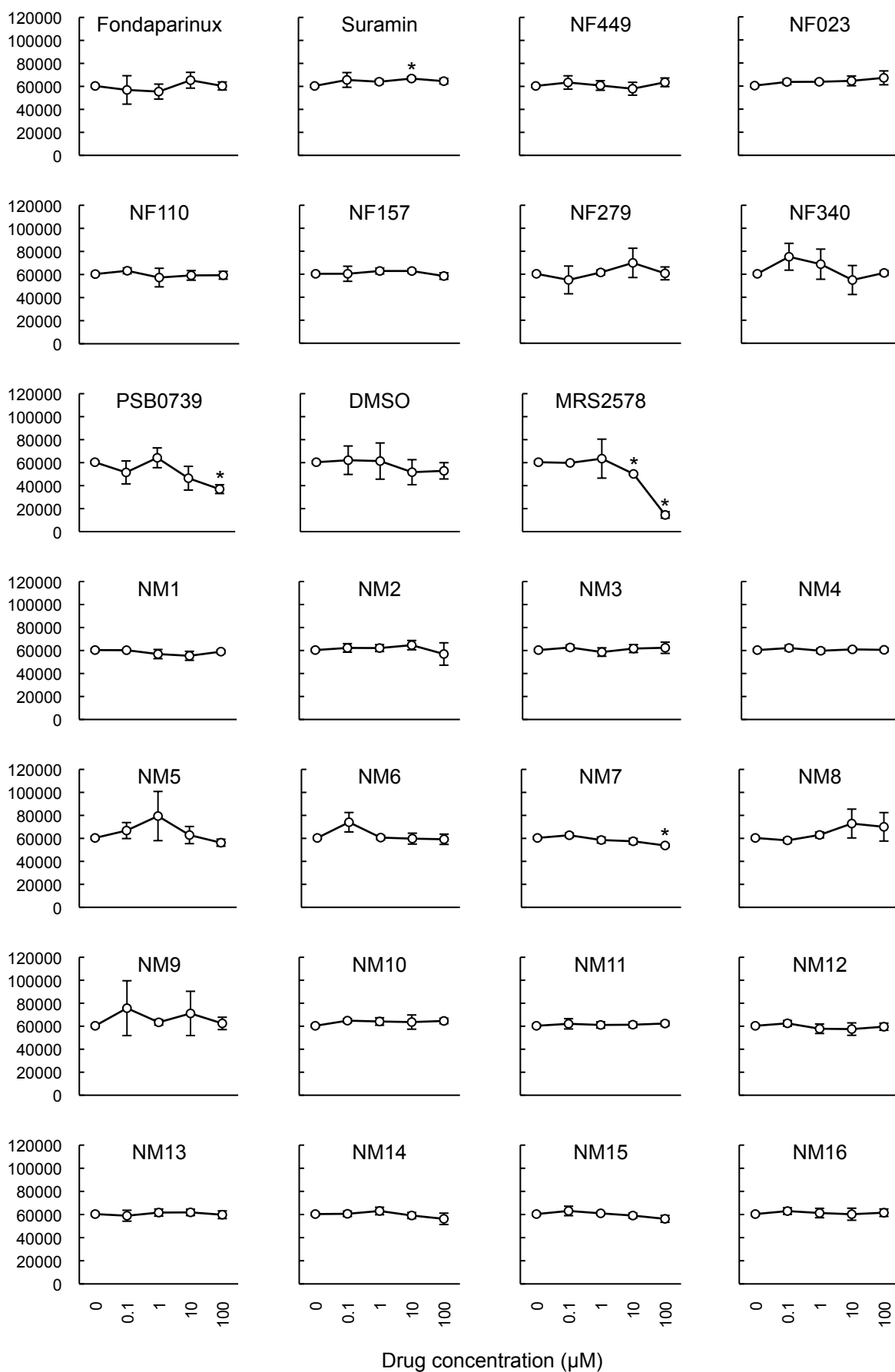

Supplement: S1 Fig — RD cells were cultured in the presence of diluted drugs in triplicate for 16 h. Cell viability was then determined by measuring a luminescent signal, which indicated the amount of ATP present, as described in Materials and Methods. (PDF) [file ppat.1005184.s001.pdf]

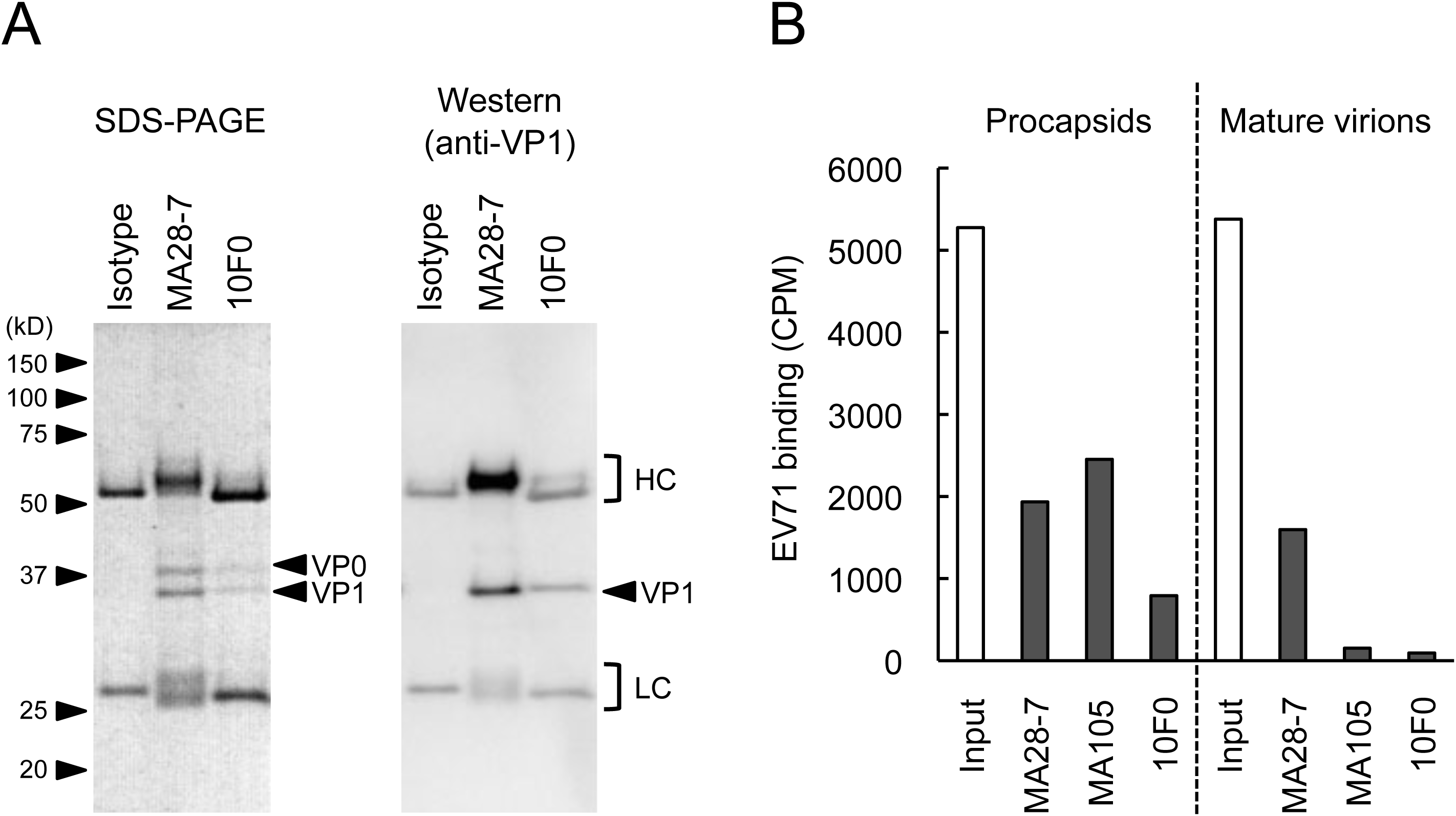

Supplement: S2 Fig — (A) Virus immunoprecipitated by MA28-7 or 10F0 was resolved by SDS-PAGE, and either stained with Coomassie blue (left panel) or transferred to membranes and stained with mouse monoclonal antibody MA105 (specific for VP1) followed by horseradish peroxidase-conjugated anti-mouse IgG antibody (right panel). (B) Aliquots of pooled gradient fractions containing 35S-labeled Procapsids or Mature virions were immunoprecipitated with MA28-7, MA105, or 10F0, and precipitates were analyzed by scintillation counting. Similar results were obtained in two independent experiments. (TIF) [file ppat.1005184.s002.tif]

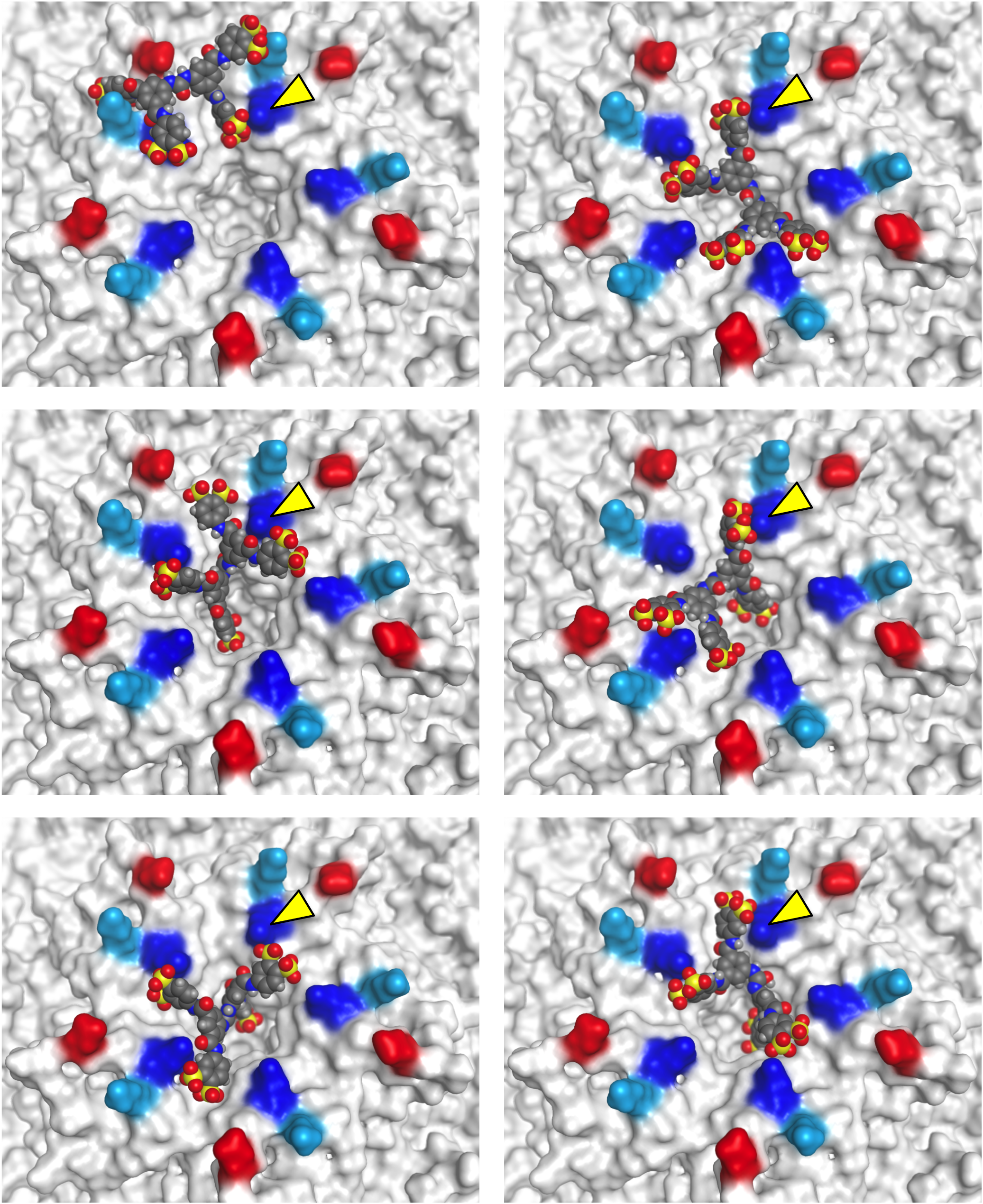

Supplement: S3 Fig — Docking sites were simulated under the constraint that one of the sulfonate groups of NF449 must interact with at least one VP1-244K residue (yellow arrowheads). The six sites with highest scores were selected from a total of 56 possible sites identified by MOE software. On the virus surface, VP1-98 is colored red, VP1-244K dark blue, and VP1-242K light blue. NF449 carbon atoms are grey, with nitrogen blue, sulfur yellow, and oxygen red. (TIF) [file ppat.1005184.s003.tif]
