# Supplementary material for: The Suramin Derivative NF449 Interacts with the 5-fold Vertex of the Enterovirus A71 Capsid to Prevent Virus Attachment to PSGL-1 and Heparan Sulfate
Source: PLoS Pathog. 2015 Oct 2;11(10):e1005184. doi: 10.1371/journal.ppat.1005184 (PMC4592248; doi:10.1371/journal.ppat.1005184)
Supplement: S1 Appendix — (DOCX) [file ppat.1005184.s004.docx]

**S1 Appendix. Methods for synthesis of compounds NM1-16.**

**General Procedures**

Solvents used for extraction and purification were HPLC grade from Fisher. Unless otherwise indicated, all reactions were run under an inert atmosphere of argon. Merck pre-coated silica gel plates (250 mm, 60 F254) were used for analytical TLC. Spots were visualized using 254 nm ultraviolet light. Chromatographic purifications were performed on Sorbent Technologies silica gel (particle size 32-63 microns). ^1^H and ^13^C NMR spectra were recorded at 500 MHz and 125 MHz, in d6-DMSO, CDCl_3_ and MeOD on a Bruker AM-500 or a DRX-500 spectrometer. Chemical shifts are reported relative to internal d6-DMSO (δ 2.54 for ^1^H), CDCl_3_ (δ 7.26 for ^1^H), MeOD (δ 3.34 for ^1^H). Infrared spectra were recorded as a solid using a Perkin-Elmer 1600 series Fourier transform spectrometer. UV/VIS spectra were obtained using a Varian-530. High resolution mass spectra were obtained at the University of Pennsylvania Mass Spectrometry Service Center on an Autospec high resolution double-focusing electrospray ionization/chemical ionization spectrometer with either DEC 11/73 or OPUS software data system. Melting points were obtained on a Thomas Hoover capillary melting point apparatus and are uncorrected.

**2,2'-((5-nitroisophthaloyl)bis(azanediyl))dibenzenesulfonate disodium salt**:

Aniline-2-sulfonic acid (6.650 g, 38.40 mmol, 2.40 equiv.) was suspended in water (154.0 mL) and the pH of the resulting mixture was adjusted to 5.0 using 2 M aqueous Na_2_CO_3_. TO the resulting solution at 25^o^C was added a solution of 5-nitroisophthalyl dichloride (3.968 g, 16.00 mmol, 1.00 equiv.) in anhydrous toluene (8.4 mL) over 2 hours via syringe pump with stirring. The internal pH of the reaction was monitored during this addition and kept at 5.0 via the addition of 2 M aqueous Na_2_CO_3_. Stirring was continued for one hour on completion of addition, at which point TLC analysis indicated completion of the reaction. The reaction mixture was extracted with EtOAc (3 × 70 mL) and these organic extracts were discarded. The aqueous layer was concentrated under reduced pressure. The resulting residue was heated to 60 ˚C in MeOH (200 mL). The resulting mixture was then filtered, and the solid product was then collected and dried to give a white solid (8.21 g, 91% yield).

R*_f_* = 0.50, reversed phase (H_2_O:MeCN; 4:1); HRMS (ES) calcd for C_20_H_13_N_3_O_10_NaS_2_ 541.9940, found 541.9956; δ_H_ (500 MHz, DMSO) 7.18 (2H, t, *J* = 7.5 Hz, ArH), 7.45 (2H, t, *J* = 7.0 Hz, ArH), 7.78 (2H, dd, *J* = 1.5, 7.5 Hz, ArH), 8.51 (2H, d, *J* = 7.5 Hz, ArH), 8.94 (3H, s, ArH), 11.94 (2H, s, NH) ppm; δ_c_ (125 MHz, DMSO) 120.5, 124.0, 124.7, 127.6, 130.5, 132.5, 134.9, 136.2, 137.3, 149.0, 161.7 ppm.

**2,2'-((5-aminoisophthaloyl)bis(azanediyl))dibenzenesulfonate disodium salt:**

To a solution of 2,2'-((5-nitroisophthaloyl)bis(azanediyl))dibenzenesulfonate disodium salt (2.071 g, 3.66 mmol, 1.00 equiv.) in water (22 mL) was added 10 wt % palladium on carbon (0.389 g, 0.37 mmol, 0.10 equiv.). The reaction was placed under a pressurised hydrogen atmosphere (500 psi) in a Parr hydrogenator. After stirring for 12 hours stirring at 25^o^C, TLC analysis showed that the reaction had reached completion. The reaction mixture was filtered on Celite and the resulting solution concentrated to give a brown solid (1.301 g, 66% yield).

R*_f_* = 0.80, reversed phase (H_2_O:MeCN; 4:1); HRMS (ES) calcd for C_20_H_15_N_3_O_8_Na_3_S_2_ 557.9994, found 558.0003; δ_H_ (500 MHz, DMSO) 5.66 (2H, s, NH_2_), 7.07 – 7.10 (2H, m, ArH), 7.28 (2H, s, ArH), 7.36 – 7.39 (2H, m, ArH), 7.60 (1H, s, ArH), 7.72 – 7.73 (2H, m, ArH), 11.31 (2H, s, NH) ppm; δ_c_ (125 MHz, DMSO) 113.7, 115.3, 120.5, 123.0, 127.4, 130.1, 135.6, 136.1, 136.9, 149.9, 164.9 ppm.

**NM2:**

**2,2',2'',2'''-((5,5'(carbonylbis(azanediyl))bis(isophthaloyl))tetrakis(azanediyl))-tetrabenzenesulfonate tetrasodium salt:**

2,2'-((5-aminoisophthaloyl)bis(azanediyl))dibenzenesulfonate disodium salt (0.677 g, 1.26 mmol, 1.00 equiv.) was dissolved in water (8 mL) and the pH of the resulting solution was adjusted to 4.0 using 2 M aqueous Na_2_CO_3_. Using a syringe pump, a solution of triphosgene (0.780 g, 2.63 mmol, 2.08 equiv.) in anhydrous toluene (4 mL) was added over 2 hours to the stirred aqueous solution. TLC analysis showed that the reaction had reached completion at the end of the addition of triphosgene. During the reaction, a solid formed which was collected via filtration. This solid was recrystallized from hot methanol and water (1:1; 25 mg per mL) to give the product as a white solid (0.69 g, 99% yield).

R*_f_* = 0.40, reversed phase (H_2_O:MeCN; 4:1; δ_H_ (500 MHz, DMSO) 7.11 – 7.14 (4H, m, ArH), 7.40 – 7.43 (4H, m, ArH), 7.75 (4H, dd, *J* = 1.5, 7.5 Hz, ArH), 8.12 (2H, s, ArH), 8.25 (4H, d, *J* = 1.0 Hz, ArH), 8.51 (4H, d, *J* = 8.0 Hz, ArH), 9.41 (2H, s, NH), 11.53 (4H, s, NH) ppm; δ_c_ (125 MHz, DMSO) 119.7, 120.2, 120.6, 123.3, 127.5, 130.2, 135.5, 136.2, 136.8, 140.9, 152.9, 164.0 ppm.

**3-(*N*-methyl-3-(methyl(4-sulfonatophenyl)carbamoyl)-5-nitrobenzamido)-benzenesulfonate disodium salt**:

*N*-methylsulfanilic acid (3.35 g, 17.87 mmol, 2.40 equiv.) was suspended in water (72.0 mL) at 25^o^C and the pH of the resulting mixture was adjusted to 5.0 using 2 M aqueous Na_2_CO_3_. To the resulting solution was added, via syringe pump, a solution of 5-nitroisophthalyl dichloride (1.85 g, 7.45 mmol, 1.00 equiv.) in anhydrous toluene (4.0 mL) over 2 hours to the stirred solution. The internal pH of the reaction was monitored during this addition and kept at 5.0 via the addition of 2 M aqueous Na_2_CO_3_. Once addition was complete, the reaction was stirred at 25^o^C for a further 1 hour. TLC analysis confirmed that the reaction had reached completion at this point. The reaction mixture was washed with EtOAc (3 × 50 mL) and these organic washings were discarded. The aqueous layer was concentrated to give a white residue. This residue was then triturated with hot MeOH (200 mL) to give, after drying the resulting solid under vacuum, a gray solid (4.42 g, 99% yield).

R*_f_* = 0.85, reversed phase (H_2_O:MeCN; 4:1); HRMS (ES) calcd. for C_22_H_17_N_3_O_10_NaS_2_ 570.0253, found 570.0238; δ_H_ (500 MHz, DMSO) 3.30 (6H, s, CH_3_), 7.08 (4H, s(br), ArrH), 7.15 – 7.16 (5H, m, ArH), 8.01 (2H, s(br), ArH) ppm; δ_C_ (125 MHz, DMSO) 37.9, 120.2, 121.7, 123.9, 126.5, 126.7, 137.5, 143.6, 146.5, 166.4 ppm.

**4,4'-((5-aminoisophthaloyl)bis(methylazanediyl))dibenzenesulfonate disodium salt**:

To a solution of 3-(*N*-methyl-3-(methyl(4-sulfonatophenyl)carbamoyl)-5-nitrobenzamido)-benzenesulfonate disodium salt (2.25 g, 3.79 mmol, 1.00 equiv.) in water (23 mL) was added 10 wt % palladium on carbon (0.40 g, 0.38 mmol, 0.10 equiv.). The reaction was placed under a pressurised hydrogen atmosphere (500 psi) in a Parr hydrogenator. After 8 hours stirring at 25^o^C, TLC analysis showed that the reaction had reached completion. The reaction mixture was filtered on celite, and the filter cake washed with MeOH (100 mL). The resulting solution was concentrated to give the product as a white solid (1.64 g, 77% yield).

R*_f_* = 0.90, reversed phase (H_2_O:MeCN; 4:1); HRMS (ES) calcd. for C_22_H_19_N_3_O_8_NaS_2_ 540.0811, found 540.0811; δ_H_ (500 MHz, DMSO) 3.20 (6H, s, CH_3_), 5.21 (2H, s(br), NH), 6.21 (1H, s, ArH), 6.42 (2H, s, ArH), 6.95 (4H, d, *J* = 8.0 Hz, ArH), 7.54 (4H, d, *J* = 8.0 Hz, ArH) ppm; δ_C_ (125 MHz, DMSO) 37.9, 114.5, 116.0, 125.7, 126.4, 136.4, 144.5, 145.6, 169.5 ppm.

**NM4:**

**4,4',4'',4'''-((5,5'-(carbonylbis(azanediyl))bis(isophthaloyl))tetrakis-(methylazanediyl))-tetrabenzenesulfonate tetrasodium salt**:

4,4'-((5-aminoisophthaloyl)bis(methylazanediyl))dibenzenesulfonate disodium salt

(0.81 g, 1.44 mmol, 1.00 equiv.) was dissolved in water (9.0 mL) and the pH of the resulting solution was adjusted to 4.0 using 2 M aqueous Na_2_CO_3_. Using a syringe pump, a solution of triphosgene (0.89 g, 2.98 mmol, 2.08 equiv.) in anhydrous toluene (4.4 mL) was added over 2 hours to the stirred aqueous solution. The reaction was stirred for a further 2 hours, whereupon ^1^H NMR analysis of an aliquot of the reaction mixture confirmed that it had gone to completion. This reaction could not be followed by TLC due to the product co-running with the starting material. The reaction mixture was then extracted with EtOAc (3 × 40 mL) to remove any unreacted triphosgene and the combined organic extracts were then stirred overnight with 5 M aqueous NaOH and methanol to quench triphosgene/phosgene. The aqueous solution was concentrated under reduced pressure to give a white solid. The solid was dissolved in MeOH (100 mL) to separate the desired product from insoluble salts. Concentration of the filtered solution and washing of the resulting solid with hot acetone (100 mL) gave the product as an off white solid (0.67 g, 81% yield).

δ_H_ (500 MHz, DMSO) 3.19 (12H, s, CH_3_), 6.57 (2H, s, ArH), 7.01 (8H, d, *J* = 7.5 Hz, ArH), 7.51 (4H, s, ArH), 7.57 (8H, d, *J* = 8.5 Hz, ArH), 11.43 (2H, s(br), NH) ppm; δ_C_ (125 MHz, DMSO) 38.4, 110.3, 118.8, 126.4, 126.9, 136.5, 140.9, 144.7, 145.8, 153.4, 169.4 ppm.

**5,5'-(carbonylbis(methylazanediyl))diisophthalic acid:**

5-(methylamino)isophthalic acid [1] (3.93 g, 16.42 mmol, 1.00 equiv.) was suspended in deionized water (100 mL). The pH of this mixture was adjusted to 4 using 2 M aqueous Na_2_CO_3_. To the resulting solution was added, with stirring via syringe pump, a solution of triphosgene (9.74 g, 32.84 mmol, 2.00 equiv.) in anhydrous toluene (40.0 mL) over 2 hours. The reaction was then stirred at 25^o^C for a further 22 hours. The reaction mixture was then acidified to pH 1 using 1 M aqueous HCl, which resulted in the precipitation of the desired product, which was then collected via filtration and washed with hot acetone (100 mL) to remove impurities. The resulting material was dried under vacuum to give the product as a white solid (2.17 g, 63% yield).

R*_f_* = 0.85, reverse phase (H_2_O:MeCN; 4:1); HRMS (ES) calcd. for C_19_H_15_N_2_O_9_ 415.0778, found 415.0776; δ_H_ (500 MHz, DMSO) 3.18 (6H, s, CH_3_), 7.54 (4H, d, *J* = 1.5 Hz, ArH), 7.97 (1H, s, ArH), 13.24 (4H, s, COOH) ppm; δ_C_ (125 MHz, DMSO) 38.8, 129.8, 132.3, 137.0, 145.6, 159.4, 166.2 ppm.

**NM5:**

**4,4',4'',4'''-((5,5'-(carbonylbis(methylazanediyl))bis(isophthaloyl))tetrakis(azanediyl))-tetrabenzenesulfonate tetrasodium salt:**

A flame-dried 3-necked round-bottomed flask was charged with 5,5'-(carbonylbis(methylazanediyl))-diisophthalic acid (0.15 g, 0.37 mmol, 1.00 equiv.), HATU (0.58 g, 1.53 mmol, 4.20 equiv.) and anhydrous DMF (6 mL). The resulting reaction was stirred at 25^o^C under an atmosphere of argon for 30 minutes before sulfanilic acid (0.27 g, 1.53 mmol, 4.20 equiv.) and then Hunig’s base (0.6 mL, 3.65 mmol, 10.00 equiv.) were added. The resulting reaction was stirred at 25^o^C for 24 hours, following which TLC analysis showed complete consumption of starting material. The reaction was then poured onto water (50 mL) and 2 M aqueous NaOH was added until the pH of the mixture reached 10. The mixture was washed with CHCl_3_ (3 × 30 mL). Combined chloroform washings were discarded at this point.

The aqueous solution was concentrated and the reside was triturated with hot MeOH (100 mL). The filtered methanolic solution was concentrated and the resulting product was washed with hot acetone (2 × 100 mL). The product was dried in an oven at 80 ˚C, to give a hard white solid (0.14 g, 35% yield).

R*_f_* = 0.65, reverse phase (H_2_O:MeCN; 4:1); δ_H_ (500 MHz, DMSO) 3.28 (6H, s, CH_3_), 7.59 (8H, d, *J* = 8.5 Hz, ArH), 7.70 (8H, d, *J* = 8.5 Hz, ArH), 7.87 (4H, d, *J* = 1.5 Hz, ArH), 8.23 (2H, s, ArH), 10.34 (4H, s(br), NH) ppm; δ_C_ (125 MHz, DMSO) 38.5, 119.6, 122.9, 124.2, 126.1, 135.6, 138.9, 143.8, 145.0, 159.4, 164.3 ppm.

**Tetramethyl 5,5'-(2-oxodihydropyrimidine-1,3(2H,4H)-diyl)diisophthalate:**

To a microwave tube was added tetrahydropyrimidin-2(1*H*)-one (0.58 g, 5.79 mmol, 1.00 equiv.), dimethyl 5-bromoisophthalate [2] (3.80 g, 13.87 mmol, 2.40 equiv.), XANTPHOS (0.33 g, 0.58 mmol, 0.10 equiv.), Pd_2_(dba)_3_·CHCl_3_ (0.30 g, 0.29 mmol, 0.05 equiv.) and Cs_2_CO_3_ (7.53 g, 23.12 mmol, 4.00 equiv.). The vial was sealed and placed under an atmosphere of argon. Dioxane (46 mL, 0.3 M) was added and the reaction was heated to 100 ˚C for 12 hours. The reaction was followed by TLC and NMR and, once complete, it was cooled to ambient temperature. The reaction mixture was filtered on Celite, and the filter cake washed with MeOH (25 mL). The resulting solution was concentrated under reduced pressure. The crude reaction material was purified via flash column chromatography [eluent (gradient): (Hex:EtOAc; 2:1) → (Hex:EtOAc; 1:1)] to give the product as a yellow solid (1.39 g, 50% yield).

R*_f_* = 0.25 (hex:EtOAc; 1:1); HRMS (ES) calcd. for C_24_H_25_N_2_O_9_ 485.1560, found 485.1565; δ_H_ (500 MHz, CDCl_3_) 2.43 – 2.39 (2H, m, CH_2_), 3.93 – 3.94 (16H, m, CH_2_, CH_3_), 8.23 (4H, d, *J* = 1.5 Hz, ArH), 8.51 (2H, t, *J* = 1.5 Hz, ArH) ppm; δ_C_ (125 MHz, CDCl_3_) 22.9, 49.0, 52.4, 127.8, 130.8, 131.2, 144.0, 153.8, 165.8 ppm.

**5,5'-(2-oxodihydropyrimidine-1,3(2*H*, 4*H*)-diyl)diisophthalic acid**:

Tetramethyl 5,5'-(2-oxodihydropyrimidine-1,3(2H,4H)-diyl)diisophthalate (1.39 g, 2.86 mmol, 1.00 equiv.) was suspended in a solution of THF (6.2 mL) and water (19.0 mL) at 25^o^C. Finely ground KOH (1.61 g, 28.61 mmol, 20.00 equiv.) was added and the resulting mixture was stirred for 12 hours. TLC analysis showed complete consumption of starting material at this point. The reaction mixture was diluted with water (50 mL) and then acidified to pH 1 using 1 M aqueous HCl to precipitate the product, which was then collected via filtration, affording a white solid (1.15 g, 94% yield).

R*_f_* = 0.65, reversed phase as COONa (H_2_O:MeCN; 4:1); HRMS (ES) calcd. for C_20_H_17_N_2_O_9_ 429.0934, found 429.0947; δ_H_ (500 MHz, DMSO) 2.22 – 2.26 (2H, m, CH_2_), 3.86 (4H, t, *J* = 6.0 Hz, CH_2_), 8.14 (4H, d, *J* = 1.5 Hz, ArH), 8.26 (2H, s, ArH), 13.39 (4H, s(br), COOH); δ_C_ (125 MHz, DMSO) 22.8, 48.9, 126.8, 130.8, 132.0, 145.0, 154.0, 166.8 ppm.

**NM6**:

**4,4',4'',4'''-((5,5'-(2-oxodihydropyrimidine-1,3(2H,4H)-diyl)bis(isophthaloyl))-tetrakis(azanediyl))tetrabenzenesulfonate tetrasodium salt:**

A flame-dried three-necked round-bottomed flask was charged with 5,5'-(2-oxodihydropyrimidine-1,3(2*H*, 4*H*)-diyl)diisophthalic acid (0.16 g, 0.38 mmol, 1.00 equiv.), TBTU (0.51 g, 1.58 mmol, 4.20 equiv.) and anhydrous DMF (6 mL, 16 mL per mmol) under an atmosphere of argon. The resulting solution was stirred at 25^o^C for 30 minutes before sulfanilic acid (0.27 g, 1.58 mmol, 4.20 equiv.) and then DIPEA (0.7 mL, 3.78 mmol, 10.00 equiv.) were added. The resulting solution was stirred at 25^o^C for 24 hours, following which TLC analysis showed complete consumption of starting material. The reaction was then poured onto water (50 mL) and 2 M aqueous NaOH was added until the pH of the mixture reached 10. The mixture was washed with CHCl_3_ (3 × 30 mL). Combined chloroform washings were discarded at this point.

The aqueous solution was concentrated and the residue was triturated with hot MeOH (100 mL) and then filtered. Concentration of the filtrate and washing the residue with hot acetone (2 × 100 mL) gave the product, which was dried in an oven at 80 ˚C to give a brown solid (0.21 g, 50% yield).

R*_f_* = 0.45, reverse phase (H_2_O:MeCN; 4:1); δ_H_ (500 MHz, DMSO) 2.31 – 2.33 (2H, m, CH_2_), 3.98 (4H, t, *J* = 5.5 Hz, CH_2_), 7.62 (8H, d, *J* = 8.5 Hz, ArH), 7.76 (8H, d, *J* = 8.0 Hz, ArH), 8.16 (4H, d, *J* = 1.0 Hz, ArH), 8.39 (2H, s, ArH), 10.52 (4H, s(br), NH) ppm; δ_C_ (125 MHz, DMSO) 22.9, 31.1, 119.8, 124.0, 126.5, 128.5, 135.7, 139.5, 143.9, 144.6, 153.9, 165.1 ppm.

**5,5'-(thiocarbonylbis(azanediyl))diisophthalic acid**:

5-aminoisophthalic acid (1.07 g, 5.88 mmol, 1.00 equiv.) was suspended in water (37.0 mL) at 25^o^C. The pH of the solution was adjusted to 5.0 using 2.0 M aqueous Na_2_CO_3_. To the resulting solution was added, using a syringe pump, a solution of thiophosgene (0.27 mL, 3.53 mmol, 0.60 equiv.) in toluene (2.0 mL) over 2 hours. Following addition, the reaction was heated to reflux and heating was maintained for 12 hours. After this, TLC analysis showed complete consumption of the starting material. The pH of the reaction mixture was then adjusted to 1 using 1 M aqueous HCl, which led to the precipitation of the product, which was collected via filtration and washed with hot acetone (200 mL) to give the product as a white solid (0.86 g, 72% yield).

R*_f_* = 0.80, reversed phase (H_2_O:MeCN; 4:1); HRMS (ES) calcd. for C_17_H_13_N_2_O_8_S 405.0393, found 405.0394; δ_H_ (500 MHz, MeOD) 8.34 (4H, d, *J* = 1.5 Hz, ArH), 8.48 (2H, d, *J* = 1.5 Hz, ArH) ppm; δ_C_ (125 MHz, MeOD) 127.1, 128.8, 133.1, 139.5, 168.4, 181.2 ppm.

**NM7**:

**4,4',4'',4'''-((5,5'-(thiocarbonylbis(azanediyl))bis(isophthaloyl))tetrakis(azanediyl))-tetrabenzenesulfonate tetrasodium salt:**

A flame-dried 3-necked round-bottomed flask was charged with 5,5'-(thiocarbonylbis(azanediyl))diisophthalic acid (0.21 g, 0.52 mmol, 1.00 equiv.), HATU (0.83 g, 2.19 mmol, 4.20 equiv.) and anhydrous DMF (8.4 mL) under an atmosphere of argon. The resulting solution was stirred at 25^o^C for 30 minutes before sulfanilic acid (0.38 g, 2.19 mmol, 4.20 equiv.) and then DIPEA (0.9 mL, 5.22 mmol, 10.00 equiv.) were added. The resulting reaction was stirred at 25^o^C for 24 hours, following which TLC analysis showed complete consumption of starting material. The reaction was then poured onto water (50 mL) and 2 M aqueous NaOH was added until the pH of the mixture reached 10. The mixture was washed with CHCl_3_ (3 × 30 mL). Combined chloroform washings were discarded at this point.

The aqueous solution was concentrated and the residue was triturated with hot MeOH (100 mL) and then filtered. Concentration of the filtrate and washing the residue with hot acetone (2 × 100 mL) gave the product.

**NM8**:

**4,4'-((3,3'-(carbonylbis(azanediyl))bis(benzoyl))bis(azanediyl))dibenzenesulfonate disodium salt:**

Under an atmosphere of argon, a flame-dried 3-necked round-bottomed flask was charged with 3,3'-(carbonylbis(azanediyl))dibenzoic acid [3] (0.20 g, 0.67 mmol, 1.00 equiv.), TBTU (0.45 g, 1.40 mmol, 2.10 equiv.) and anhydrous DMF (10.6 mL). The resulting reaction was stirred at 25^o^C for 30 minutes before sulfanilic acid (0.23 g, 1.35 mmol, 2.02 equiv.) and then DIPEA (0.6 mL, 3.33 mmol, 5.00 equiv.) were added. The resulting reaction was stirred at 25^o^C for 24 hours, following which TLC analysis showed complete consumption of starting material. The reaction was then poured onto water (50 mL) and 2 M aqueous NaOH was added until the pH of the mixture reached 10. The mixture was washed with CHCl_3_ (3 × 30 mL). Combined chloroform washings were discarded at this point.

The aqueous solution was concentrated and the product was washed with hot MeOH (200 mL). The product was dried in an oven at 80 ˚C, to give a brown solid (0.39 g, 89% yield).

R*_f_* = 0.25, reverse phase (H_2_O:MeCN; 4:1); HRMS (ES) calcd. for C_27_H_19_N_4_O_9_Na_2_S_2_ 653.0389, found 653.0375; δ_H_ (500 MHz, DMSO) 7.44 (2H, t, *J* = 8.0 Hz, ArH), 7.55 – 7.60 (6H, m, ArH), 7.73 (4H, d, *J* = 8.5 Hz ArH), 7.77 (2H, d, *J* = 8.5 Hz, ArH), 7.98 (2H, s, ArH), 9.30 (2H, s(br), NH), 10.33 (2H, s(br), NH) ppm; δ_C_ (125 MHz, DMSO) 117.8, 119.2, 121.0, 121.3, 126.0, 128.8, 135.7, 139.3, 140.0, 143.5, 152.7, 165.7 ppm.

**NM9**:

**4,4'-((3,3'-(carbonylbis(azanediyl))bis(benzoyl))bis(azanediyl))dibenzenesulfonate disodium salt:**

Under argon, a flame-dried 3-necked round-bottomed flask was charged with 4,4'-(carbonylbis(azanediyl))dibenzoic acid [3] (0.20 g, 0.67 mmol, 1.00 equiv.), TBTU (0.45 g, 1.40 mmol, 2.10 equiv.) and anhydrous DMF (10.6 mL). The resulting reaction was stirred at 25^o^C for 30 minutes before sulfanilic acid (0.23 g, 1.35 mmol, 2.02 equiv.), then DIPEA (0.6 mL, 3.33 mmol, 5.00 equiv.) were added. The resulting reaction was stirred at 25^o^C for 24 hours, following which TLC analysis showed complete consumption of starting material. The reaction was then poured onto water (50 mL) and 2 M aqueous NaOH was added until the pH of the mixture reached 10. The mixture was washed with CHCl_3_ (3 × 30 mL). Combined chloroform washings were discarded at this point.

The aqueous solution was concentrated and the residue was triturated with hot MeOH (200 mL), which on concentration and drying in an oven at 80 ˚C gave an off-white solid (0.09 g, 20% yield).

R*_f_* = 0.25, reverse phase (H_2_O:MeCN; 4:1); HRMS (ES) calcd. for C_27_H_19_N_4_O_9_Na_2_S_2_ 653.0389, found 653.0367; δ_H_ (500 MHz, DMSO) 7.57 (2H, d, *J* = 8.0 Hz, ArH), 7.62 (2H, d, *J* = 8.0 Hz, ArH), 7.72 (2H, d, *J* = 8.0 Hz, ArH), 7.85 (2H, d, *J* = 8.0 Hz, ArH), 9.20 (2H, s(br), NH), 10.15 (2H, s(br), NH) ppm; δ_C_ (125 MHz, DMSO) 117.8, 119.7, 126.4, 128.4, 129.3, 139.8, 143.1, 144.0, 152.6, 165.4 ppm.

**NM11**:

**4,4'-((5-(3-phenylureido)isophthaloyl)bis(azanediyl))dibenzenesulfonate disodium salt:**

At 25^o^C, a solution of 4,4'-(5-aminoisophthaloylbisimino)-bis(benzenesulfonic acid) disodium salt (0.15 g, 0.28 mmol, 1.00 equiv.) was dissolved in THF (4 mL) and water (1 mL). Phenyl Isocyanate (128.0 μL, 0.56 mmol, 4.00 equiv.) was added to the reaction mixture and the resulting solution was then stirred for 16 hours until complete consumption of starting material was observed by TLC. The reaction was concentrated to remove THF and the resulting residue was partitioned between water (10 mL) and DCM (10 mL). The layers were separated and the aqueous layer was further washed with DCM (2 × 10 mL). Combined organic washings were discarded and the aqueous layer was concentrated to dryness. The resulting solid was washed with hot acetone (20 mL) and then triturated with hot MeOH (20 mL). The filtered methanolic solution was concentrated to give the product as a brown solid (0.09 g, 50% yield).

R*_f_* = 0.40, reverse phase (H_2_O:MeCN; 4:1); δ_H_ (500 MHz, DMSO) 6.99 (1H, t, *J* = 7.5 Hz, ArH), 7.30 (2H, t, *J* = 8.0 Hz, ArH), 7.50 (2H, d, *J* = 8.0 Hz, ArH), 7.59 (4H, d, *J* = 8.5 Hz, ArH), 7.75 (4H, d, *J* = 8.5 Hz, ArH), 8.15 – 8.17 (3H, m, ArH), 8.86 (1H, s(br), NH), 9.18 (1H, s(br), NH), 10.48 (2H, s(br), NH) ppm.

**Dimethyl 5-(3-(3-(methoxycarbonyl)phenyl)ureido)isophthalate**:

A solution of phosgene in toluene (15wt %, 20 mL, 26.50 mmol, 4.02 equiv.) was placed in a 2-necked round-bottomed flask equipped with a reflux condenser and heated to 80 ˚C. Using a syringe pump, a solution of methyl 3-aminobenzoate (1.00 g, 6.60 mmol, 1.00 equiv.) was added to the reaction mixture over 3 hours. The reaction was then stirred for a further 2 hours and cooled to 25^o^C. This reaction mixture was concentrated under reduced pressure and used for the second operation without further purification.

The residue isolated was dissolved in dichloromethane (50 mL) and dimethyl 5-aminoisophthalate (1.11 g, 5.28 mmol, 0.8 equiv.) was added. The resulting reaction was then stirred at 25^o^C for 12 hours. Following this, the reaction mixture was washed with a saturated aqueous solution of NH_4_Cl (3 × 35 mL), then brine (50 mL). The organic solution was dried over Na_2_SO_4_ and concentrated to give a brown liquid. This liquid was purified via flash column chromatography [eluent (gradient): (hexane:EtOAc; 4:1) → (hexane:EtOAc; 2:1)] to give the product as a white solid (0.20 g, 8% yield).

R*_f_* = 0.05 (hex:EtOAc; 2:1); HRMS (ES) calcd. for C_19_H_17_N_2_O_7_ 385.1036, found 385.1035; δ_H_ (500 MHz, DMSO) 3.86 (3H, s, CH_3_), 3.89 (6H, s, CH_3_), 7.44 (1H, t, *J* = 8.0 Hz, ArH), 7.59 (1H, dt, *J* = 1.0, 7.5 Hz, ArH), 7.66 (1H, ddd, *J* = 1.0, 2.5, 8.0 Hz, ArH), 8.09 (1H, t, *J* = 1.5 Hz, ArH), 8.21 (1H, t, *J* = 2.0 Hz, ArH), 8.34 (2H, d, *J* = 1.5 Hz, ArH), 9.02 (1H, s(br), NH), 9.27 (1H, s(br), NH) ppm; δ_C_ (125 MHz, DMSO) 52.6, .53.0, 119.5, 123.3 (3C), 123.4, 129.7, 130.6, 131.1, 140.2, 141.1, 152.9, 165.8, 166.6 ppm.

**5-(3-(3-(carboxyphenyl)ureido)isophthalic acid**:

At 25^o^C, dimethyl 5-(3-(3-(methoxycarbonyl)phenyl)ureido)isophthalate (0.24 g, 0.61 mmol, 1.00 equiv.) was suspended in THF (1.3 mL) and water (4.0 mL). Finely ground KOH (0.69 g, 12.24 mmol, 20.00 equiv.) was added and the reaction was stirred for 12 hours. TLC analysis showed complete consumption of starting material at this point. The reaction mixture was diluted with water (10 mL) and then acidified to pH 1 using 1 M aqueous HCl, which resulted in precipitation of the product. The product was then collected via filtration and dried in an oven at 80 ˚C, affording a brown solid (0.04 g, 21% yield).

R*_f_* = 0.45, reversed phase as COONa (H_2_O:MeCN; 4:1); HRMS (ES) calcd. for C_20_H_17_N_2_O_9_ 429.0934, found 429.0947; δ_H_ (500 MHz, DMSO) 7.41 (1H, t, *J* = 8.0 Hz, ArH), 7.57 (1H, dd, *J* = 1.0, 7.5 Hz, ArH), 7.68 (1H, d, *J* = 8.0 Hz, ArH), 8.10 (1H, d, *J* = 1.5 Hz, ArH), 8.11 (1H, d, *J* = 1.5 Hz, ArH), 8.30 (2H, d, *J* = 1.5 Hz, ArH), 9.18 (1H, s(br), NH), 9.38 (1H, s(br), NH), 13.10 (3H, s(br), COOH) ppm; δ_C_ (125 MHz, DMSO) 119.6, 123.1, 123.2, 123.4, 123.8, 129.5, 131.8, 132.2, 140.2, 140.9, 153.0, 167.0, 167.7 ppm

**NM12**:

**4,4'-((5-(3-(3-((4-sulfonatophenyl)carbamoyl)phenyl)ureido)isophthaloyl)bis-(azanediyl))dibenzenesulfonate trisodium salt:**

Under an argon atmosphere, a flame-dried three-necked round-bottomed flask was charged with 5-(3-(3-(carboxyphenyl)ureido)isophthalic acid (25.0 mg, 0.07 mmol, 1.00 equiv.), TBTU (75.0 mg, 0.23 mmol, 3.20 equiv.) and anhydrous DMF (1.2 mL, 16 mL per mmol). The resulting reaction was stirred at 25^o^C for 30 minutes before sulfanilic acid (41.0 mg, 0.23 mmol, 3.20 equiv.) and then DIPEA (0.1 mL, 0.73 mmol, 8.00 equiv.) were added. The resulting reaction was stirred at 25^o^C for 24 hours, following which TLC analysis showed complete consumption of starting material. The reaction was then poured onto water (10 mL) and 2 M aqueous NaOH was added until the pH of the mixture reached 10. The mixture was washed with CHCl_3_ (3 × 10 mL). Combined chloroform washings were discarded at this point.

The aqueous solution was concentrated and the product was triturated with hot MeOH (30 mL). The product was dried in an oven at 80 ˚C to give a brown solid (23.0 mg, 36% yield). R*_f_* = 0.40, reverse phase (H_2_O:MeCN; 4:1); δ_H_ (500 MHz, DMSO) 7.43 (1H, t, *J* = 8.0 Hz, ArH), 7.54 – 7.58 (7H, m, ArH), 7.70 – 7.72 (6H, m, ArH), 7.86 – 7.89 (2H, m, ArH), 8.12 (1H, s, ArH), 8.20 (2H, s, ArH), 9.50 (1H, s(br), NH), 9.65 (1H, s(br), NH), 10.28 (1H, s(br), NH), 10.44 (1H, s(br), NH) ppm; δ_C_ (125 MHz, DMSO) 119.8, 119.9, 121.2 (2C), 126.6, 128.7, 136.4, 139.7, 139.8, 144.3, 144.5, 153.2, 153.4, 165.9 ppm.

**4,4'-((5-nitroisophthaloyl)bis(azanediyl))bis(naphthalene-1-sulfonate) disodium salt:**

At 25^o^C, 4-aminonaphthalene-1-sulfonic acid (6.48 g, 29.03 mmol, 2.40 equiv.) was suspended in water (117.0 mL) and the pH of the resulting mixture was adjusted to 5.0 using 2 M aqueous Na_2_CO_3_. To the resulting solution was added, using a syringe pump, a solution of 5-nitroisophthalyl dichloride (3.00 g, 12.10 mmol, 1.00 equiv.) in anhydrous toluene (6.4 mL) over 2 hours. A precipitate was observed. The internal pH of the reaction was monitored during this addition and kept at 5.0 via the addition of 2 M aqueous Na_2_CO_3_. Once addition was complete, the reaction was stirred at 25^o^C for a further 1 hour. TLC analysis confirmed that the reaction had reached completion at this point. The precipitate was collected via filtration and washed with hot acetone (200 mL). The precipitate was then dried overnight in an oven (80 ˚C) to give the product as a white solid (7.63 g, 95% yield).

R*_f_* = 0.60, reversed phase (H_2_O:MeCN; 4:1); m/z ES^-^ (M – Na; 100%); δ_H_ (500 MHz, DMSO) 7.56 – 7.61 (6H, m, ArH), 8.02 (2H, d, *J* = 7.5 Hz, ArH), 8.07 – 8.09 (2H, m, ArH), 8.92 – 8.94 (2H, m, ArH), 9.11 (2H, s, ArH), 9.24 (2H, s, ArH), 11.01 (2H, s(br), NH) ppm; δ_C_ (125 MHz, DMSO) 122.8, 123.6, 124.6, 125.8, 126.1, 126.2, 128.5, 129.8, 130.3, 133.8, 148.5, 164.3 ppm.

**4,4'-((5-aminoisophthaloyl)bis(azanediyl))bis(naphthalene-1-sulfonate) disodium salt:**

To a solution of 4,4'-((5-nitroisophthaloyl)bis(azanediyl))bis(naphthalene-1-sulfonate) disodium salt (2.40 g, 3.60 mmol, 1.00 equiv.) in water (21.6 mL) was added 10 wt % palladium on carbon (0.38 g, 0.36 mmol, 0.10 equiv.). The reaction was placed under a pressurised hydrogen atmosphere (500 psi) in a Parr hydrogenator. After 12 hours stirring at 25^o^C, TLC analysis showed that the reaction had reached completion. The reaction mixture was filtered on Celite, and the filter pad washed with MeOH (100 mL). Concentration of the methanolic solution gave a white solid (1.06 g, 46% yield).

R*_f_* = 0.75, reversed phase (H_2_O:MeCN; 4:1); HRMS (ES) calcd. for C_28_H_19_N_3_NaO_8_S_2_^-^ 612.0517, found 612.0501; δ_H_ (500 MHz, DMSO) 5.63 (2H, s(br), NH), 7.44 (2H, s, ArH), 7.53 – 7.55 (6H, m, ArH), 7.98 (2H, s, ArH), 8.00 (1H, s, ArH), 8.04 – 8.06 (2H, m, ArH), 8.90 – 8.92 (2H, m, ArH), 10.41 (2H, s(br), NH) ppm; δ_C_ (125 MHz, DMSO) 114.6, 116.5, 122.6, 123.7, 124.6, 125.9, 126.1, 128.4, 129.9, 130.3, 135.4, 136.1, 142.8, 149.6, 167.1 ppm.

**NM13**:

**4,4',4'',4'''-((5,5'-(carbonylbis(azanediyl))bis(isophthaloyl))tetrakis(azanediyl))-tetrakis(naphthalene-1-sulfonate) tetrasodium salt:**

4,4'-((5-aminoisophthaloyl)bis(azanediyl))bis(naphthalene-1-sulfonate) disodium salt (0.43 g, 0.68 mmol, 1.00 equiv.) was dissolved in water (5 mL) and the pH of the resulting solution was adjusted to 4.0 using 2 M aqueous Na_2_CO_3_. The resulting solution was then heated to 80 ˚C and this temperature was maintained throughout the reaction. Using a syringe pump, a solution of triphosgene (0.30 g, 0.16 mmol, 1.50 equiv.) in anhydrous toluene (2.0 mL) was added over 2 hours to the stirred solution. Following addition, the reaction was stirred for a further 3 hours. The internal pH of the reaction mixture was maintained at 4.0, via addition of 2.0 M aqueous Na_2_CO_3_.

The resulting reaction mixture was cooled to 25^o^C and 2.0 M aqueous NaOH (0.5 mL) was added. The reaction mixture was then extracted with EtOAc (3 × 40 mL) to remove any unreacted triphosgene and the combined organic extracts were then stirred overnight with 5 M aqueous NaOH and methanol to destroy residual triphosgene/phosgene. The aqueous solution was concentrated under reduced pressure. The resulting residue was triturated with a mixture of hot acetone and MeOH (1:1; 200 mL). The remaining solid was dried in an oven at 80 ˚C for 4 hours to give the product as an off-white solid (0.32 g, 73% yield).

R*_f_* = 0.75, reversed phase (H_2_O:MeCN; 4:1); δ_H_ (500 MHz, DMSO) 7.56 – 7.59 (12H, m, ArH), 8.02 (4H, d, *J* = 7.5 Hz, ArH), 8.10 (4H, s, ArH), 8.42 (4H, s, ArH), 8.57 (2H, s, ArH), 8.92 (4H, d, *J* = 4.5 Hz, ArH), 10.30 (2H, s(br), NH), 10.74 (4H, s(br), NH) ppm; δ_C_ (125 MHz, DMSO) 121.1, 121.4, 122.7, 123.8, 124.7, 126.0, 126.2, 128.3, 129.9, 130.2, 135.4, 136.0, 140.8, 142.6, 153.4, 166.5 ppm.

**5,5'-((5-nitroisophthaloyl)bis(azanediyl))bis(naphthalene-1-sulfonate) disodium salt:**

At 25^o^C, 5-aminonapthalene-1-sulfonic acid (6.48 g, 29.03 mmol, 2.40 equiv.) was suspended in water (117.0 mL) and the pH of the resulting mixture was adjusted to 5.0 using 2 M aqueous Na_2_CO_3_. To the resulting solution was added, using a syringe pump, a solution of 5-nitroisophthalyl dichloride (3.00 g, 12.10 mmol, 1.00 equiv.) in anhydrous toluene (6.4 mL) over 2 hours. The internal pH of the reaction was monitored during this addition and kept at 5.0 via the addition of 2 M aqueous Na_2_CO_3_. Once addition was complete, the reaction was stirred at 25^o^C for a further 1 hour. TLC analysis confirmed that the reaction had reached completion at this point. The resulting precipitate was collected via filtration and washed with hot acetone (200 mL). The precipitate was then dried overnight in an oven (80 ˚C) to give the product as a grey solid (4.55 g, 57% yield).

R*_f_* = 0.60, reversed phase (H_2_O:MeCN; 4:1); HRMS (ES) calcd. for C_28_H_16_N_3_O_10_Na_2_S_2_ 664.0073, found 664.0056; δ_H_ (500 MHz, DMSO) 7.49 – 7.52 (2H, m, ArH), 7.58 – 7.64 (4H, m, ArH), 8.02 (2H, d, *J* = 7.0 Hz, ArH), 8.07 (2H, d, *J* = 8.5 Hz, ArH), 8.87 (2H, d, *J* = 8.5 Hz, ArH), 9.12 (2H, s, ArH), 9.25 (1H, s, ArH), 11.02 (2H, s(br), NH) ppm; δ_C_ (125 MHz, DMSO) 124.4, 125.1, 125.2, 125.6, 125.8, 127.2, 130.2, 130.3, 133.6, 136.8, 144.7, 148.5, 164.3 ppm.

**5,5'-((5-aminoisophthaloyl)bis(azanediyl))bis(naphthalene-1-sulfonate) disodium salt:**

5,5'-((5-nitroisophthaloyl)bis(azanediyl))bis(naphthalene-1-sulfonate)

To a solution of 5,5'-((5-nitroisophthaloyl)bis(azanediyl))bis(naphthalene-1-sulfonate) disodium salt (2.56 g, 3.55 mmol, 1.00 equiv.) in water (21.0 mL) was added 10 wt % palladium on carbon (0.37 g, 0.35 mmol, 0.10 equiv.). The reaction was placed under a pressurised hydrogen atmosphere (500 psi) in a Parr hydrogenator. After 12 hours stirring at 25^o^C, TLC analysis showed that the reaction had reached completion. The reaction mixture was filtered on Celite, and the filter pad washed with MeOH (100 mL). Evaporation under reduced pressure gave a brown solid (2.00 g, 89% yield).

R*_f_* = 0.75, reversed phase (H_2_O:MeCN; 4:1); m/z ES^-^ [M – Na (612.0417); 40%]; δ_H_ (500 MHz, DMSO) 5.64 (2H, s(br), NH), 7.46 (2H, s, ArH), 7.48 – 7.51 (2H, m, ArH), 7.56 – 7.58 (4H, m, ArH), 8.00 – 8.05 (5H, m, ArH), 8.83 (2H, dd, *J* = 2.5, 7.0 Hz, ArH), 10.44 (2H, s(br), NH) ppm; δ_C_ (125 MHz, DMSO) 114.7, 116.5, 124.3, 124.9, 125.1, 125.4, 125.6, 126.6, 130.3, 130.4, 134.3, 136.1, 144.5, 149.6. 167.2 ppm.

**NM14**:

**5,5',5'',5'''-((5,5'-(carbonylbis(azanediyl))bis(isophthaloyl))tetrakis(azanediyl))-tetrakis(naphthalene-1-sulfonate) tetrasodium salt:**

5,5'-((5-aminoisophthaloyl)bis(azanediyl))bis(naphthalene-1-sulfonate) disodium salt (0.63 g, 0.99 mmol, 1.00 equiv.) was dissolved in water (6 mL) and the pH of the resulting solution was adjusted to 4.0 using 2 M aqueous Na_2_CO_3_. The resulting solution was then heated to 80 ˚C and this temperature was maintained throughout the reaction. Using a syringe pump, a solution of triphosgene (0.44 g, 1.49 mmol, 1.50 equiv.) in anhydrous toluene (5.0 mL) was added over 2 hours to the stirred solution. Following addition, the reaction was stirred for a further 3 hours. The internal pH of the reaction mixture was maintained at 4.0, via addition of 2.0 M aqueous Na_2_CO_3_.

The resulting reaction mixture was cooled to 25^o^C and 2.0 M aqueous NaOH (0.5 mL) was added. The reaction mixture was then extracted with EtOAc (3 × 40 mL) to remove any unreacted triphosgene and combined organic extracts were then stirred overnight with 5 M aqueous NaOH and methanol to discharge unreacted triphosgene/phosgene. The aqueous solution was concentrated under reduced pressure to give a brown residue. The resulting residue was triturated with a mixture of hot acetone and MeOH (1:1; 200 mL). The material obtained was dried in an oven at 80 ˚C for 4 hours, to give the product as an off-white solid (0.31 g, 48% yield).

R*_f_* = 0.75, reversed phase (H_2_O:MeCN; 4:1); δ_H_ (500 MHz, DMSO) 7.48 (4H, t, *J* = 7.5 Hz, ArH), 7.54 – 7.60 (8H, m, ArH), 7.99 (4H, d, *J* = 7.0 Hz, ArH), 8.07 (4H, d, *J* = 8.5 Hz, ArH), 8.39 (4H, s, ArH), 8.60 (2H, s, ArH), 8.83 (4H, d, *J* = 8.5 Hz, ArH), 8.32 (2H, s(br), NH), 10.74 (4H, s(br), NH) ppm; δ_C_ (125 MHz, DMSO) 120.5, 123.8, 124.4, 124.6, 125.0, 125.1, 126.3, 129.8, 129.9, 133.7, 135.5, 140.6, 144.1, 150.3, 166.1 ppm.

**NM15**:

**5,5',5'',5'''-((5,5'-(carbonylbis(azanediyl))bis(isophthaloyl))tetrakis(azanediyl))-tetrakis(naphthalene-2-sulfonate) tetrasodium salt:**

5,5'-((5-aminoisophthaloyl)bis(azanediyl))bis(naphthalene-2-sulfonate) disodium salt disodium salt (0.70 g, 1.10 mmol, 1.00 equiv.) was dissolved in water (7 mL) and the pH of the resulting solution was adjusted to 4.0 using 2 M aqueous Na_2_CO_3_. The resulting solution was then heated to 80 ˚C and this temperature was maintained throughout the entire reaction. Using a syringe pump, a solution of triphosgene (0.49 g, 1.65 mmol, 1.50 equiv.) in anhydrous toluene (2.5 mL) was added over 2 hours to the stirred solution. Following addition, the reaction was stirred for a further 3 hours. The internal pH of the reaction mixture was maintained at 4.0, via addition of 2.0 M aqueous Na_2_CO_3_.

The resulting reaction mixture was cooled to 25^o^C and 2.0 M aqueous NaOH (0.5 mL) was added. The reaction mixture was then extracted with EtOAc (3 × 40 mL) to remove any unreacted triphosgene and combined organic extracts were then stirred overnight with 5 M aqueous NaOH and methanol to destroy unreacted triphosgene/phosgene. The aqueous solution was concentrated under reduced pressure to give a dark red residue. The resulting residue was triturated with MeOH (200 mL). The material obtained was dried in an oven at 80 ˚C for 4 hours, to give the product as a wine-colored solid (0.50 g, 71% yield).

R*_f_* = 0.70, reversed phase (H_2_O:MeCN; 4:1); δ_H_ (500 MHz, DMSO) 7.60 – 7.66 (8H, m, ArH), 7.78 (4H, s(br), ArH), 7.94 (4H, s(br), ArH), 8.05 (4H, s(br), ArH), 8.23 (4H, s(br), ArH), 8.41 (4H, s(br), ArH), 8.57 (2H, s(br), ArH), 10.18 (2H, s(br), NH), 10.75 (4H, s(br), ArH) ppm; δ_C_ (125 MHz, DMSO) 120.5, 120.7, 123.4, 123.9, 124.4, 126.1, 127.0, 128.9, 133.0, 133.8, 135.5, 140.3, 145.4, 153.0, 166.1 ppm.

**5,5'-(carbonylbis(azanediyl))bis(N^1^,N^3^-bis(4-hydroxyphenyl)isophthalamide):**

Under an argon atmosphere, a flame-dried 3-necked round-bottomed flask was charged with 5,5'-ureylenediisophthalic acid (1.02 g, 2.64 mmol, 1.00 equiv.), HATU (4.21 g, 11.07 mmol, 4.20 equiv.) and anhydrous DMF (40.0 mL). The resulting reaction was stirred at 25^o^C for 30 minutes before 4-aminophenol (1.21 g, 11.07 mmol, 4.20 equiv.), then DIPEA (4.6 mL, 26.35 mmol, 10.00 equiv.) were added. The resulting reaction was stirred at 25^o^C for 24 hours, following which TLC analysis showed complete consumption of starting material. The reaction was then poured onto water (160 mL) which resulted in the precipitation of a green solid. This solid contained both product and HOAt (by-product from using HATU). HOAt was washed away from the product using boiling water (150 mL). The product was obtained as a brown solid (1.13 g, 57% yield) after drying in an over at 80 ˚C.

R*_f_* = 0.65 (EtOAc:MeOH; 4:1); HRMS (ES) calcd. for C_41_H_32_N_6_O_9_Na 775.2128, found 775.2130; δ_H_ (500 MHz, DMSO) 6.76 (8H, d, *J* = 9.0 Hz, ArH), 7.56 (8H, d, *J* = 9.0 Hz, ArH), 8.11 (2H, s, ArH), 8.17 (4H, s, ArH), 9.24 (2H, s(br), NH), 9.27 (4H, s(br), OH), 10.18 (4H, s(br), NH) ppm; δ_C_ (125 MHz, DMSO) 115.5, 120.4, 120.8, 122.6, 131.1, 136.6, 140.3, 153.1, 154.2, 165.1 ppm.

**NM16**:

**5,5'-(carbonylbis(azanediyl))bis(N^1^,N^3^-bis(4-(((λ^1^-oxidanyl)dioxo-λ^6^-sulfanyl)oxy)-phenyl)isophthalamide) tetrasodium salt:**

5,5'-(carbonylbis(azanediyl))bis(N^1^,N^3^-bis(4-hydroxyphenyl)isophthalamide) (0.07 g, 0.09 mmol, 1.00 equiv.), acetonitrile (0.5 mL, 9 mL per g), DMF (0.13 mL), trimethylamine (0.5 mL, 3.76 mmol, 40.00 equiv.) and trimethylamine-sulfur trioxide complex (0.79 g, 5.64 mmol, 60.00 equiv.) were combined in a microwave tube. The tube was sealed and heated to 100 ˚C in a microwave reactor for 40 minutes. The resulting mixture was cooled to 25^o^C and filtered to remove excess trimethylamine-sulfur trioxide complex. The filtrate was then concentrated.

The resulting residue was taken up in water (25 mL) and washed with CHCl_3_ (3 × 25 mL). The aqueous layer was concentrated to give a white solid. This solid was triturated with hot MeOH (40 mL) and the resulting methanolic solution was concentrated to give a solid which was washed with hot acetone (40 mL). The product was isolated as a off-white solid after drying in an oven at 80 ˚C (0.08 g, 77% yield).

δ_H_ (500 MHz, DMSO) 7.13 (8H, dd, *J* = 9.5, 11.5 Hz, ArH), 7.65 – 7.67 (8H, m, ArH), 8.13 (2H, s, ArH), 8.20 (4H, s, ArH), 9.46 (2H, s(br), NH), 10.33 (4H, s(br), NH) ppm; δ_C_ (125 MHz, DMSO) 120.7, 121.2, 121.3, 121.7, 13.0, 136.6, 140.6, 150.3, 153.3, 165.5 ppm.

**References for S1 Appendix:**

1. Wentworth P, Datta A, Smith S, Marshall A, Partridge LJ, Blackburn GM. Antibody Catalysis of BAc2 Aryl Carbamate Ester Hydrolysis:  A Highly Disfavored Chemical Process. Journal of the American Chemical Society. 1997;119(9):2315-6.

2. Okada Y, Yokozawa M, Akiba M, Oishi K, O-kawa K, Akeboshi T, et al. Bromination by means of sodium monobromoisocyanurate (SMBI). Organic & Biomolecular Chemistry. 2003;1(14):2506-11.

3. Drewe WC, Nanjunda R, Gunaratnam M, Beltran M, Parkinson GN, Reszka AP, et al. Rational Design of Substituted Diarylureas: A Scaffold for Binding to G-Quadruplex Motifs. Journal of medicinal chemistry. 2008;51(24):7751-67.
